# Prevalence and practices of immunofluorescent cell image processing: a systematic review

**DOI:** 10.3389/fncel.2023.1188858

**Published:** 2023-07-20

**Authors:** Hawley Helmbrecht, Teng-Jui Lin, Sanjana Janakiraman, Kaleb Decker, Elizabeth Nance

**Affiliations:** ^1^Department of Chemical Engineering, University of Washington, Seattle, WA, United States; ^2^Paul G. Allen School of Computer Science & Engineering, Seattle, WA, United States; ^3^Department of Bioengineering, University of Washington, Seattle, WA, United States

**Keywords:** image processing, brain cells, fluorescent imaging, cell biology, thresholding, segmentation, data science

## Abstract

**Background:**

We performed a systematic review that identified at least 9,000 scientific papers on PubMed that include immunofluorescent images of cells from the central nervous system (CNS). These CNS papers contain tens of thousands of immunofluorescent neural images supporting the findings of over 50,000 associated researchers. While many existing reviews discuss different aspects of immunofluorescent microscopy, such as image acquisition and staining protocols, few papers discuss immunofluorescent imaging from an image-processing perspective. We analyzed the literature to determine the image processing methods that were commonly published alongside the associated CNS cell, microscopy technique, and animal model, and highlight gaps in image processing documentation and reporting in the CNS research field.

**Methods:**

We completed a comprehensive search of PubMed publications using Medical Subject Headings (MeSH) terms and other general search terms for CNS cells and common fluorescent microscopy techniques. Publications were found on PubMed using a combination of column description terms and row description terms. We manually tagged the comma-separated values file (CSV) metadata of each publication with the following categories: animal or cell model, quantified features, threshold techniques, segmentation techniques, and image processing software.

**Results:**

Of the almost 9,000 immunofluorescent imaging papers identified in our search, only 856 explicitly include image processing information. Moreover, hundreds of the 856 papers are missing thresholding, segmentation, and morphological feature details necessary for explainable, unbiased, and reproducible results. In our assessment of the literature, we visualized current image processing practices, compiled the image processing options from the top twelve software programs, and designed a road map to enhance image processing. We determined that thresholding and segmentation methods were often left out of publications and underreported or underutilized for quantifying CNS cell research.

**Discussion:**

Less than 10% of papers with immunofluorescent images include image processing in their methods. A few authors are implementing advanced methods in image analysis to quantify over 40 different CNS cell features, which can provide quantitative insights in CNS cell features that will advance CNS research. However, our review puts forward that image analysis methods will remain limited in rigor and reproducibility without more rigorous and detailed reporting of image processing methods.

**Conclusion:**

Image processing is a critical part of CNS research that must be improved to increase scientific insight, explainability, reproducibility, and rigor.

## 1. Introduction

Immunofluorescent imaging is one of the most common ways to acquire cell images and quantify cell features (Im et al., 2019). A 2021 study showed that over 22% of figures from a PubMed search included some form of a photograph, which often were microscopy or fluorescent images (Jambor et al., 2021). A 2020 study analyzing image methods in biomedical research noted that image processing methods are rarely published in detail (Marqués et al., 2020). In central nervous system (CNS) research, fluorescent images are commonly used to make discoveries in cellular and subcellular mechanisms (Im et al., 2019), disease progression (Barretto et al., 2011; Lin et al., 2018), and brain mapping (Kerman, 2008), among many others. However, as we demonstrate in the results of our systematic review, < 10% of papers using fluorescent images of the central nervous system (CNS) mention image processing to support image analysis of CNS cells.

Once images are acquired with a fluorescent microscopy technique, image processing most commonly occurs in two ways: image enhancements for qualitative visualization or applied algorithms for cell feature quantification. Examples of cell feature quantification include measures for staining intensity, cell count, or specific cell area. To increase the interpretability of cell feature analyses, image processing techniques necessitate detailed methodologies for reproducible, unbiased, accurate, and high throughput cell image analyses. Since even a simple brightness enhancement is often necessary to visualize cells, all publications, including immunofluorescent images, should publish the appropriate image processing parameters; otherwise, results of quantified cell features can be significantly skewed (Lee and Kitaoka, 2018). However, publications using immunofluorescent cell imaging often include minimal detail about image processing methodologies, often disregarding brightness adjustments, image processing algorithms, and image acquisition parameters in written methodologies. Researchers recognize issues related to the reproducibility and repeatability of immunofluorescent and immunohistochemical images (Bennett et al., 2009; Lee and Kitaoka, 2018; Manuel et al., 2018; Miura and Norrelykke, 2021), yet there is little to no literature that provides an in-depth assessment of image processing practices and methods reporting in CNS research.

**Figure 1:**
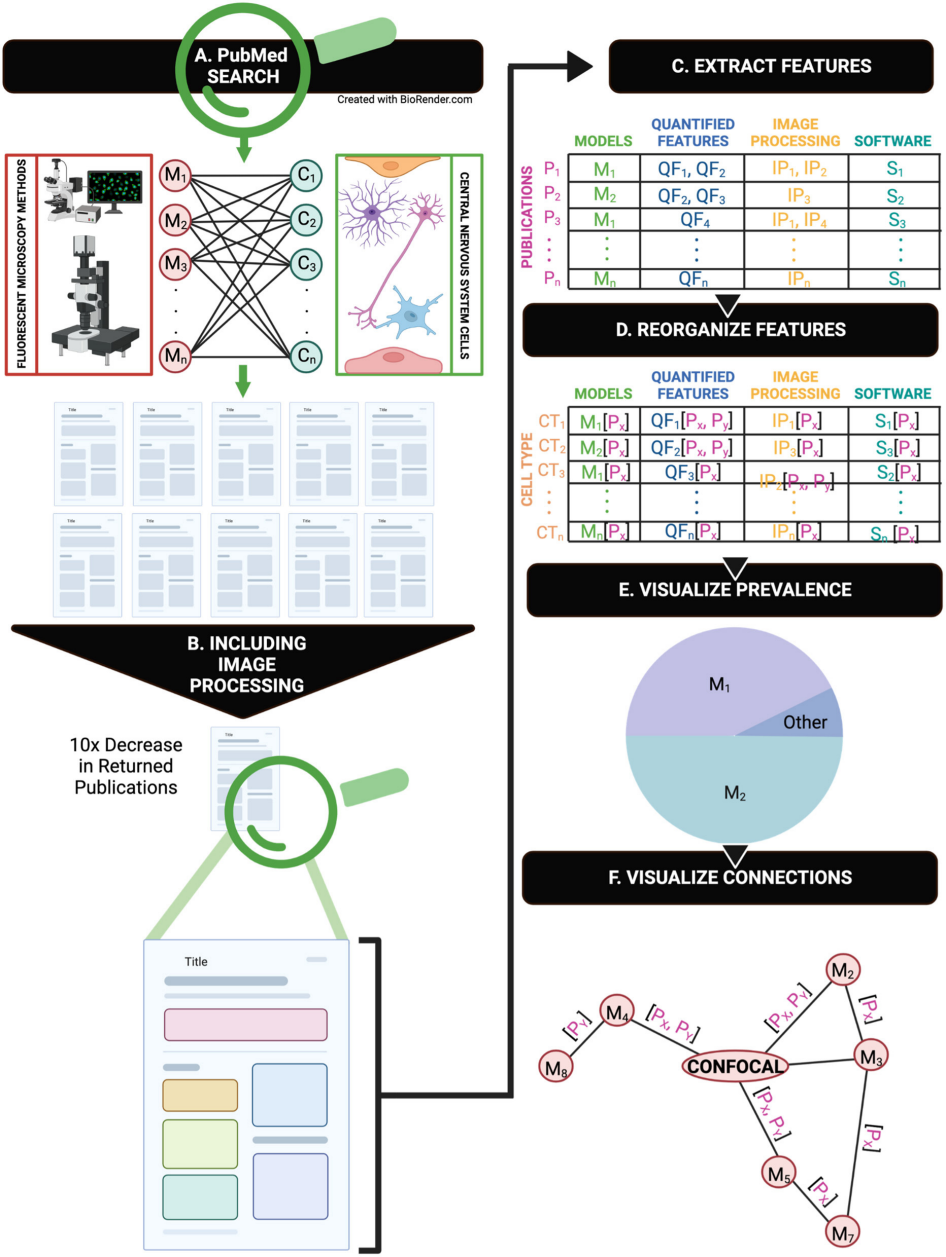
A graphical representation of the overall methodology of this review. **(A)** An overview of the PubMed search CNS cells and fluorescent microscopy combinations in publications. **(B)** A repeated search from part A with additional image processing terms returns 1/10^th^ of the original search. **(C)** A representation of manual feature extraction from each publication. **(D)** Feature rearrangement and organization by cell type rather than specific publication after manual extraction. **(E)** Reorganization of features that enables visualization of feature prevalence and connections via multiple microscopy methods.

We provide a review of the current state of image processing practices in CNS research. We first highlight a severe underreporting of image processing methods (Figure 1). In PubMed, we mined CNS papers that use fluorescent imaging (Figure 1A) and then further constrained our search to return papers that included any reference to image processing (Figure 1B). To obtain digestible insights from the mined papers, we tagged features from each paper including animal model, software, and quantified cell features (Figure 1C). We created visualizable groupings by reorganizing our tagged variables to be sorted by cell type rather than by publication, which enables cross-publication comparison of methods (Figure 1D).

Our high-level visualization of the major CNS cells—neurons, glia, and vascular cells—with common immunofluorescent imaging techniques shows the widespread application of each imaging method in CNS research (Figure 1E). As immunofluorescent imaging and computational analysis methods grow in popularity, this review provides a timely guide for improved reporting of immunofluorescent image acquisition, processing, and publishing to address existing methodological gaps. To facilitate a move to more rigorous reporting of image processing methods for immunofluorescent images, we provide a road map to guide researchers in documenting image processing steps.

## 2. Methodology

### 2.1. Literature search

We completed a comprehensive search of PubMed publications using Medical Subject Headings (MeSH) terms and other general search terms for CNS cells and common fluorescent microscopy techniques (Supplementary Tables 1, 2)—[MeSH] beside the term indicates a MeSH term; otherwise, the term is a general use term. For each section of the table, publications were found on PubMed using a combination of column description terms and row description terms. For the first search, we combined terms for different types of CNS cells with terms for fluorescent microscopy using the Boolean operator AND. The PubMed search terms used for cells: “Motor Neurons” [MeSH], “Sensory Receptor Cells” [MeSH], “Interneurons” [MeSH], “Multipolar”, “Neurons” [MeSH] AND “Multipolar”, “Neurons” [MeSH] AND “Unipolar”, “Neurons” [MeSH] AND “Bipolar”, “Neurons” [MeSH] AND “Pseudo-unipolar”, “Microglia” [MeSH], “Astrocytes” [MeSH], “Oligodendrocytes” [MeSH], “Ependymal Cells” [MeSH], “Endothelial Cells” [MeSH], “Pericytes” [MeSH], and “Muscle, Smooth Vascular” [MeSH]. All cell terms were individually combined with the search terms for different types of fluorescent microscopy: “Microscopy, Confocal” [MeSH], “Microscopy, Fluorescence” [MeSH] AND “General Fluorescent Microscopy”, “Microscopy, Fluorescence” [MeSH] AND “Widefield”, “Microscopy, Fluorescence, Multiphoton” [MeSH], “Microscopy, Fluorescence” [MeSH] AND “Light Sheet”, “Microscopy, Fluorescence” [MeSH] AND “Total Internal Reflection”, “Microscopy, Fluoresence” [MeSH] AND “Super Resolution”. For search consistency, no additional terms were added to clarify motor neuron and sensory receptor neuron location in the CNS or peripheral nervous system (Supplementary Tables 1–3).

A set of additional search terms were added for the combination of all cell search terms with the terms “Microscopy, Confocal” [MeSH] and the “Microscopy Fluorescent” [MeSH] AND “General Fluorescent Microscopy”. We first repeated the confocal and general fluorescent microscopy searches with all cell types and another search term “Image Processing, Computer-Assisted” [MeSH]. Then we repeated the search with either “Threshold” or “Segment” terms added to the search. Finally, all of the cell search terms with the “Microscopy, Confocal” [MeSH] and the “Microscopy Fluorescent” [MeSH] AND “General Fluorescent Microscoy” search combination were searched with the simple term “image” and either “threshold” or “segment”.

A comma-separated value (CSV) of all returned publications for each search was saved and included as supplemental information available on Zenodo with the https://doi.org/10.5281/zenodo.7651627. Zenodo files are saved in the format “Date_supplementary table #_Search Term1 _Boolean_Search Term N” and contain the PMID, publication title, Authors, Citation, First Author, Journal/Book, Publication Year, Creation Date, PMCID, NIHMS ID, and DOI for each returned paper.

### 2.2. Feature tagging

With a start date of January 1^st^, 2010, we separated all publications returned from the literature search using: (1) the MeSH term for confocal microscopy, (2) the CNS cell word or term, (3) and the MeSH term for image processing. The year 2010 was selected since the Fiji distribution of ImageJ–the most popularly used image processing software—was first publicly presented in 2010 and published in 2012 (Schindelin et al., 2012); at this time, computational image processing techniques were becoming more frequent, shown by a peak in the publication count in Supplementary Figure 1. We then manually tagged the CSV metadata of each publication with the following categories: animal or cell model, quantified features, threshold techniques, segmentation techniques, and image processing software. We compiled a table of each CNS cell (rows of the table) and, under each category (columns of the table), included all tagged features and references to related publications. After compiling the table, we alphabetized each cell (Supplementary Table 3).

### 2.3. Treemaps

We used Excel to create treemaps, diagrams of hierarchical data that apply nested rectangles to represent the numerical value of the unique group. Each treemap is sized so the boxes scale down from the largest group, represented by the Sensory neurons imaged with confocal microscopy that include the MeSH term for image processing.

### 2.4. Sankey diagram

Sankey diagrams were created with Plotly in Python. The number of papers tagged with a specific feature determines the size of the nodes. The links–the paths between nodes—are sized for the number of papers tagged that include the features at both ends of that link. The unspecified or unmentioned feature categories and links were colored yellow in Plotly.

### 2.5. Software comparison table

Software programs included in more than one publication are described in Table 1. Thirty-nine of the 52 software programs were only cited once within the search. Of the other 13 software, ImageJ—including the FIJI distribution—was the most common, followed by Imaris, MATLAB, Amira, and Adobe Photoshop. With Adobe Photoshop, we only considered citations that explicitly stated that Adobe Photoshop was used to adjust features of images, including but not exclusionary to features such as brightness, contrast, and color adjustment. We did not include Adobe Photoshop if it was only used for figure creation without image adjustment.

**Table 1:** Summary of commonly used software including costs and basic image processing features.

**Software name**	**Developer/parent company**	**# of papers**	**Cost**	**Thresholding methods**	**Segmentation methods**	**Quantified cell features**
ImageJ	National Institutes of Health	*56*	*Free*	Options: Global, Local, Auto (global histogram-derived) Threshold. Auto Options: Huang, Intermodesl, IsoData, Li, MaxEntropy, Mean, MinError(l), Minimum, Moments, Otsu, Percentile, RenyiEntropy, Shanbhag, Triangle, Yen (ImageJ Docs Release 1.17.2)	Options: Multiple Methods: Trainable Weka Segmentation, Auto Thresholding, and Masking, Manual Segmentation (ImageJ Docs Release 1.17.2)	Area (Debertin et al., 2015; Hannibal et al., 2017), Cell Density (Virgone-Carlotta et al., 2013; Jawaid et al., 2018), Count (Huang et al., 2012; Li et al., 2013; Ferber and Tiram, 2014; Bendali et al., 2015; Debertin et al., 2015; Sakita et al., 2016; Seidl and Rubel, 2016; Van Der Woude and Smid, 2016; Žygelyte et al., 2016; Banerjee and Chaturvedi, 2017; Hannibal et al., 2017; Wang et al., 2017; Jawaid et al., 2018; Santos et al., 2018), Colocalization (Alcami and Marty, 2013; Li et al., 2013; Virgone-Carlotta et al., 2013; Banerjee and Chaturvedi, 2017; Sousa-Valente et al., 2017), Dendritic Diameter (Jawaid et al., 2018), Diameter (Sakita et al., 2016; Seidl and Rubel, 2016; Hannibal et al., 2017), Feret's Diameter (Herron and Miles, 2012), Intensity (Dibaj et al., 2010; Fernández-Alvarez et al., 2011; Masuda et al., 2011; Smith et al., 2011; Hovis et al., 2012; Tapia et al., 2012; Trouche et al., 2013; Sousa-Valente et al., 2017; Awadová et al., 2018; Chu et al., 2020), Length (Seidl and Rubel, 2016; Van Der Woude and Smid, 2016), Location (Chu et al., 2020), Nuclei Count (Trouche et al., 2013), Perimeter (Sakita et al., 2016), Process (branch/dendrite) Count (Masuda et al., 2011; Santos et al., 2018), Process (branch/dendrite) Length (Masuda et al., 2011; Awadová et al., 2018; Santos et al., 2018), Process Extension (Masuda et al., 2011), Puncta Size (Alcami and Marty, 2013), Puncta Intensity (Alcami and Marty, 2013), Puncta Circularity (Alcami and Marty, 2013), Thickness of Processes (branches/dendrites) (Sakita et al., 2016; Tavares et al., 2017), Total Length of Processes (branches/dendrites) (Tavares et al., 2017), Sholl Analysis (Tavares et al., 2017; Santos et al., 2018), Spheroid quantification (Martin et al., 2013), Surface Area (Cain et al., 2011; Nagel et al., 2012; Azaripour et al., 2018), Volume (Goldsmith et al., 2010; Cain et al., 2011; Heinze et al., 2013; Van Der Woude and Smid, 2016; Awadová et al., 2018)
Imaris	Oxford Instruments	23	$13,000–$45,000^*^	Options: threshold cutoff, baseline subtraction, background subtraction, connective baseline (Imaris Reference Manual V 5.5.0)	Options: FilamentTracer or Segmentation “Wizards: (1) AutoPaths (no loops) for a tree-like filament or Threshold (loops). (Imaris Reference Manual V 5.5.0)	Area (Bayerl et al., 2016), Branch count (Althammer et al., 2020), Branch Ends (Huang et al., 2021), Branch Order (Huang et al., 2021), Cell Distribution Profiles Across Regions (Paul et al., 2014), Colocalization (Pihlaja et al., 2008; Sosa et al., 2013; Testen et al., 2020), Count (Choi et al., 2010; Sosa et al., 2013; Bendali et al., 2015; Bayerl et al., 2016; Miller and Rothstein, 2016; Huang et al., 2021), Density (Bayerl et al., 2016), Distance between Cells (Huang et al., 2021), Filament Length (Althammer et al., 2020), Intensity (Bayerl et al., 2016), Intersections (Dando et al., 2019), Length (Choi et al., 2010; Huang et al., 2021), Location (Choi et al., 2010), Sholl Analysis (Althammer et al., 2020), Surface Area (Althammer et al., 2020), Volume (Azaripour et al., 2018; Althammer et al., 2020)
MATLAB	Mathworks	22	$50 base	Coding language	Coding language	Coding language
Amira	Thermo Scientific	20	$4,000 + (Estimate)	Options: auto-thresholding or multi-thresholding. specific algorithms are not stated. (users guide to Amira software 2019)	Options: multi-thresholding (users guide to Amira software 2019)	Area (Williams et al., 2010), Cell coverage (Williams et al., 2010), Colocalization (Virgone-Carlotta et al., 2013), Count (Choi et al., 2010; Kelber et al., 2010; Elliott et al., 2015), Density (Virgone-Carlotta et al., 2013), Distance between cells (Williams et al., 2010), Length (Choi et al., 2010; Kelber et al., 2010; Virgone-Carlotta et al., 2013), Process (Branch/Dendrite) Count (Elliott et al., 2015), Process (Branch/Dendrite) intersections (Elliott et al., 2015), Sholl Radius (Virgone-Carlotta et al., 2013), Volume (Kelber et al., 2010; Heinze et al., 2013)
Adobe Photoshop	Adobe Inc.	15	$240/year	Options: A threshold filter option based on a user input single value (Adobe Photoshop Elements User Guide Updated in 2021).	Options: segmentation is possible with a user-created workflow (Adobe Photoshop Elements User Guide Updated in 2021)	Image adjustments were made with Adobe Photoshop, but no features were quantified.
Zeiss ZEN	Zeiss	8	$2,611 per license with basic modules	Options: manual, histogram based, size, shape, intensity, and ROI based (Zen Blue Image Analysis Guide 2016).	Options: Thresholding-Based (Zen Blue Image Analysis Guide 2016)	Colocalization (Sousa-Valente et al., 2017), Intensity (Sousa-Valente et al., 2017)
MetaMorph	Molecular Devices	7	$6,000 + base price, $750 + yearly upgrades, $1,200 offline analysis cost	Options: thresholding available in region of interest selection, Authothreshold, watershed (Fundamentals of MemaMorph Workshop Slides)	Options: watershed segmentation (Fundamentals of MemaMorph Workshop Slides)	Area coverage (Williams et al., 2010; Naguib et al., 2012), Colocalization (Li et al., 2013), Count (Choi et al., 2010; Li et al., 2013; Bolea et al., 2014), Distance between cells (Williams et al., 2010), Intensity (Ghiretti and Paradis, 2011; Naguib et al., 2012), Length (Choi et al., 2010), Synapse Density (Ghiretti and Paradis, 2011)
Volocity	PerkinElmer	7	$6,230 base	Options: manual threshold, automatic threshold (OTSU method). (Volocity User Guide V 22.0)	Volocity user guide does not include the term “segment” or “segmentation.” (Volocity User Guide V 22.0)	Area (Bayerl et al., 2016), Axon length (Kemp et al., 2016), Cell density (Bayerl et al., 2016), Colocalization (Ugbode et al., 2014; Wang et al., 2017), Count (Bayerl et al., 2016; Kemp et al., 2016), Intensity (Bayerl et al., 2016; Chu et al., 2020), Size (Kemp et al., 2016), Surface Area (Bagheri et al., 2015), Volume (Bagheri et al., 2015; Bayerl et al., 2016; Chu et al., 2020)
Image-Pro Plus	Media Cybernetics	4	$5,775	Options: manual threshold, automatic threshold (OTSU method). (Image-Pro Plus Version 7.0)	Options: manual threshold based segmentation and watershed algorithm. (Image-Pro Plus Version 7.0)	Axon length (Kemp et al., 2016), Colocalization (Kemp et al., 2016), Count (Kemp et al., 2016), Vessel diameter (Tan et al., 2013)
Avizo	Thermo Scientific	3	$10,000 +	Options: manual thresholding, auto thresholding (choose criterion based suited for data generally factorization), hysteresis thresholding (Thermo Scientific Avizo Software 9 User Guide).	Options: manual, threshold-based image segmentation with multi-thresholding or interactive thresholding, interactive top-hat (Thermo Scientific Avizo Software 9 User Guide).	Segmentation was completed with Avizo, but no quantified features were attributed to Avizo.
AutoQuant X3	Media Cybernetics	2	$7,000	Options: manual threshold, maximum/minimum thresholding (Media Cybernetics, Inc. User Manual Version X2)	OptiHistogram-BasedBased Segmentation (Media Cybernetics, Inc. User Manual Version X2)	Branch Ends (Huang et al., 2021), Branch Order (Huang et al., 2021), Count (Huang et al., 2021), Distance between Cells (Huang et al., 2021), Length (Huang et al., 2021)
Python	Python Software Foundation	2	Free	Coding language	Coding language	Coding language
Reconstruct	–	2	Custom software	–	–	–

Most commonly, image processing software programs are different than the acquisition software programs used with the specific confocal microscope that captured these images. Discussion of acquisition software programs is outside of the scope of the review, and acquisition software programs are typically dependent on the specific company for the microscopy instrument. We refer the reader to Hng and Dormann's paper about automatic confocal microscope performance evaluation (Hng and Dormann, 2013), which discusses acquisition software programs in the context of reproducibility and standardization. For the papers explored to build **Figure 3**, those with the MeSH terms “Microscopy, Confocal” and “Image Processing, Computer-Aided” published since 2010, a total of 43 unique software programs or coding languages were used. The range of software programs used only for confocal fluorescent images of CNS cells shows the large number of software programs that do exist and can provide quantitative insights into cellular morphology and features.

All associated quantified features were compiled into a table for every software program tagged (Supplementary Tables 3, 4). The different software programs and coding languages were sorted by the total number of publications mentioning the specific software program used for cell feature quantification. The software program costs were found either on the website or through email inquiry; we included a note if an email inquiry was necessary. Details for costing vary depending on the information available without obtaining a quote. Thresholding and segmentation techniques for each software were found in the appropriate user manual and using a “ctrl + find” method for “threshold” and “segment.” Quantified features and associated references were found during feature tagging. A limitation of our study is that some papers use multiple software programs without clarifying which software programs produced which results. In the case of multiple software programs in one publication without clarification, we tagged the feature to each software program used for image processing in that specific publication.

One limitation we noted is the MeSH term PubMed search left out many papers citing some of the more popular software. For example, the authors use image processing implemented in Python in their own work to study microglia morphology with fluorescent confocal microscopy (Joseph et al., 2020; Wood et al., 2021; Nguyen et al., 2022), yet none of the author's own papers were returned in the search. As another example, CellProfiler is a popular, open-source cell analysis platform that only got one citation through our search, yet, when we completed a PubMed search of all papers citing the CellProfiler software, over one hundred papers were returned (Mcquin et al., 2018). Most of the papers returned include the phrase “CellProfiler” in the title or abstract, alluding to the use of the platform being a major purpose of the paper. While our CellProfiler search does exhibit limitations of our MeSH term labeling approach of PubMed papers, our review still demonstrates a representation of the use of image processing documentation in papers analyzing immunofluorescent cells of the CNS. Possibilities for the search limitation include journal-specific tagging, field-dependent tagging or reporting, lack of reporting within publications, and differences in what is defined as “image processing” in the field.

## 3. Prevalence of fluorescent cell imaging methods

The quality of the images acquired via fluorescent microscopy affects image processing and image analysis, particularly quantification of image or cell features. In our review, we focus on papers that use immunofluorescent imaging achieved through antibody-based staining protocols to label specific target antigens with a fluorescent dye. Several recent reviews discuss the optimization of staining parameters and stain selection, including Farhoodi et al. (2019), and other reviews discuss optimizing imaging parameters and image quality for different microscopy methods (Jost and Waters, 2019; Boehm et al., 2021; Larsen et al., 2023). We will briefly focus on understanding the wide variety of fluorescent microscopes, which is helpful in grasping the complexities of cellular image processing. The most popular fluorescent imaging techniques for imaging cells of the CNS are general fluorescent microscopy, confocal microscopy, fluorescent widefield microscopy, multiphoton microscopy, light sheet microscopy, total internal reflection microscopy (TIRF), and super-resolution microscopy (Richardson, 2017). The various fluorescent microscopy techniques offer advantages and disadvantages concerning economic cost, resolution, and imaging of specific cellular and tissue structures, but cost and accessibility is often a deciding factor in which instrumentation is used. Although outside the scope of this review, we refer the reader to Renz (2013), who provides an excellent history of fluorescent microscopy, Hammer et al. (2021) who published community-driven metadata approaches for light microscopy, and Sanderson et al. (2014) who published a comprehensive review for comparison and contrast of fluorescent microscopy techniques.

**Figure 2:**
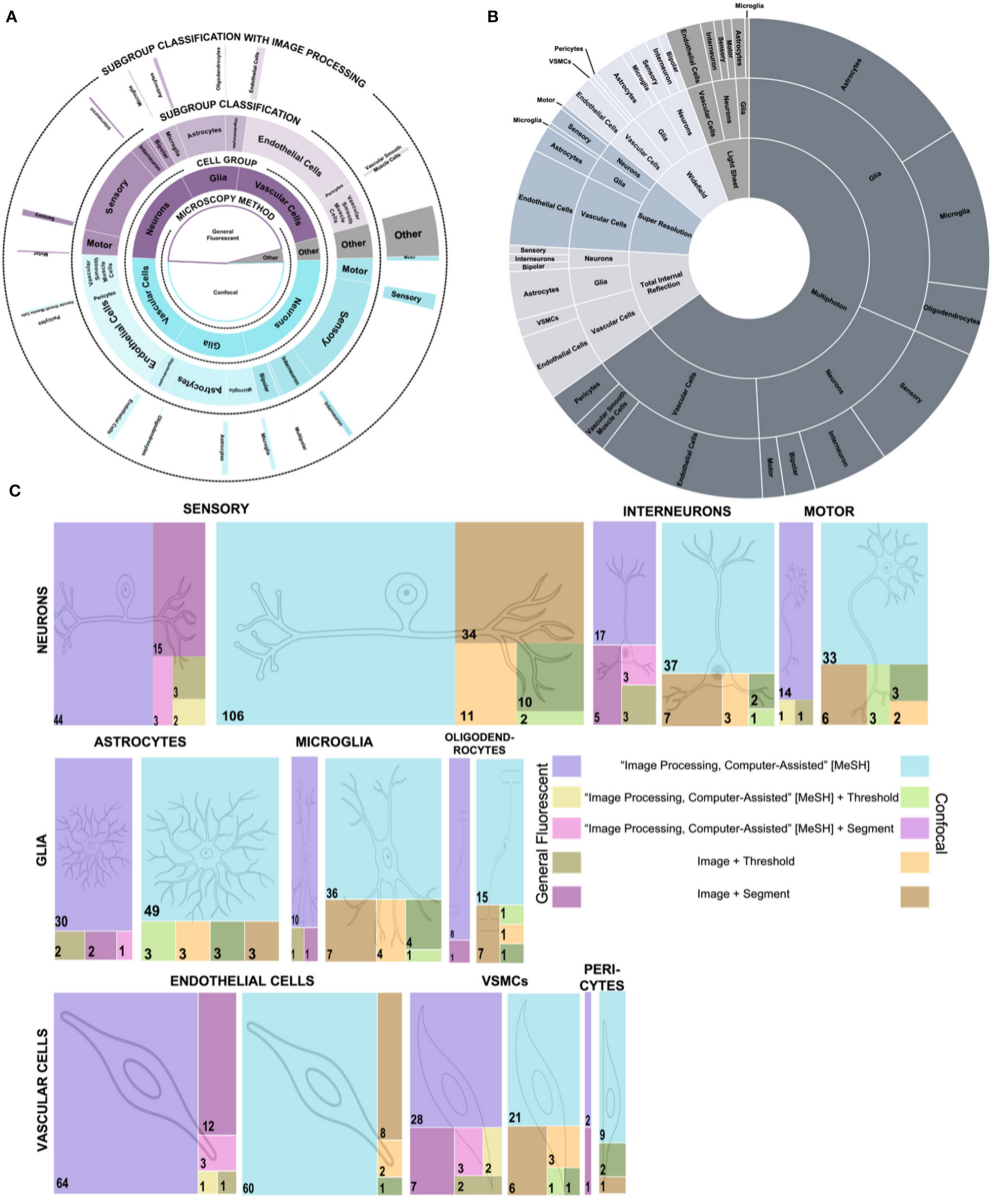
Publication prevalence of image processing across microscopy techniques and cell types. **(A)** A multi-level pie chart showing the breakdown of paper count for general fluorescent and confocal microscopy vs. others. The inner layer shows the paper count for each microscopy type. The middle layers show the cell group and sub-group classification counts for each microscopy type. The outer layer shows the number of papers for each type, including image processing. **(B)** A sunburst chart of the “Other” category from **(A)**. The paper count determines the size of the sections of the inner layer for each microscopy type. The size of the middle layer sections shows the paper count by cell group. Finally, the outer layer shows the paper count by sub-group classification. **(C)** Treemaps depict the publication count for each cell group and sub-group cell classification for different combinations of image processing terms. The left side of the key depicts the colors used for the general fluorescent microscopy publications, and the right side depicts the colors used for the confocal microscopy publications. Each cell sub-group classification has a general fluorescent and confocal microscopy treemap.

We performed a quantitative assessment of the prevalence of different fluorescent microscopy techniques for imaging CNS cells. We assessed the PubMed library for different combinations of CNS cells and fluorescent microscopy. To explore major patterns across cells and microscopy techniques, we organized our findings into a Supplementary Table 1 of publication prevalence and a multi-level pie chart (Figures 2A, B). From the papers returned in our search, confocal microscopy is the most prevalent fluorescent imaging technique for CNS cells, exceeding general fluorescent microscopy publications by about 500 total publications—a 13% difference. Together, the combination of confocal and general fluorescent microscopy comprise over 96% of all CNS papers using immunofluorescent cell imaging. For easier reference, we broke the remaining 4% of articles into a separate pie chart (Figure 2B), where multiphoton is the most prevalent type.

Across all microscopy types, we noted three general outcomes: First, endothelial cells and vascular smooth muscle cells are imaged more often with general fluorescent microscopy. Second, when looking at the sub-classifications of neurons, it is about ten times more common for neurons to be published with their functional classification than their morphological classification, regardless of microscopy type. The only exception is the bipolar neuron which has one hundred more publications than any other morphological classification. Third, out of the 9,000 evaluated articles, endothelial cells, followed by astrocytes, are the cells most broadly imaged by all fluorescent imaging techniques. Astrocytes ranked third in our search for total immunofluorescent publications yet showed the broadest range of immunofluorescent microscopy techniques used to image these cells. Astrocytes may exhibit a wider spread of published fluorescent microscopy methods since they have a complex architecture targeted uniquely with different microscopies (Barcia et al., 2013).

Beyond breaking down the immunofluorescent imaging trends of different cells of the CNS, the outer layer of our multi-level pie chart (Figure 1A) shows the sparsity of immunofluorescent papers that include image processing. Of the 8,845 articles returned in our search for CNS cells with immunofluorescent imaging, only 856 also included image processing. The 90% decrease in papers indicates an area for improvement to either publish more detailed methods about image processing or gain more information from images with image processing techniques. Intrigued by the gap in published CNS papers with immunofluorescent imaging and image processing, we created treemaps of papers published with different combinations of search terms (Figure 1C). The largest group in each treemap is the group that includes the type of microscopy, the sub-group cell classification, and the MeSH term for image processing. Our treemaps show that sensory neurons imaged with confocal microscopy and tagged with the MeSH term for image processing have the most publications. However, the number of publications that include a searchable term for threshold or segment, two commonly used image processing methods, sharply drops off for all groups, with most cell sub-group classifications having < 10 total papers including image processing terms.

The pie charts and treemaps highlight trends that could benefit from more exploration or better publication database labeling. For example, combining multiple microscopy techniques to image astrocyte and endothelial cells could enable deeper analysis of unique aspects of the cells, cell subpopulations, or subcellular components. At the same time, our visualized data provides opportunities to researchers to identify areas of cell research that may benefit from further exploration with new microscopy techniques. While the authors believe this provides a holistic look at the field, limitations to the search method include that the papers must be in the PubMed catalog and that papers must be searchable by specific tags. For improved searchability, databases like PubMed could add new categorical search terms, additional labeling techniques, and improved back-labeling. To improve the use of image analysis via, for example, moving from semi-quantitative to quantitative analysis of CNS cells, researchers need documented and detailed current image processing methodologies for immunofluorescent images of CNS cells.

## 4. Current image processing applications on CNS cells

**Figure 3:**
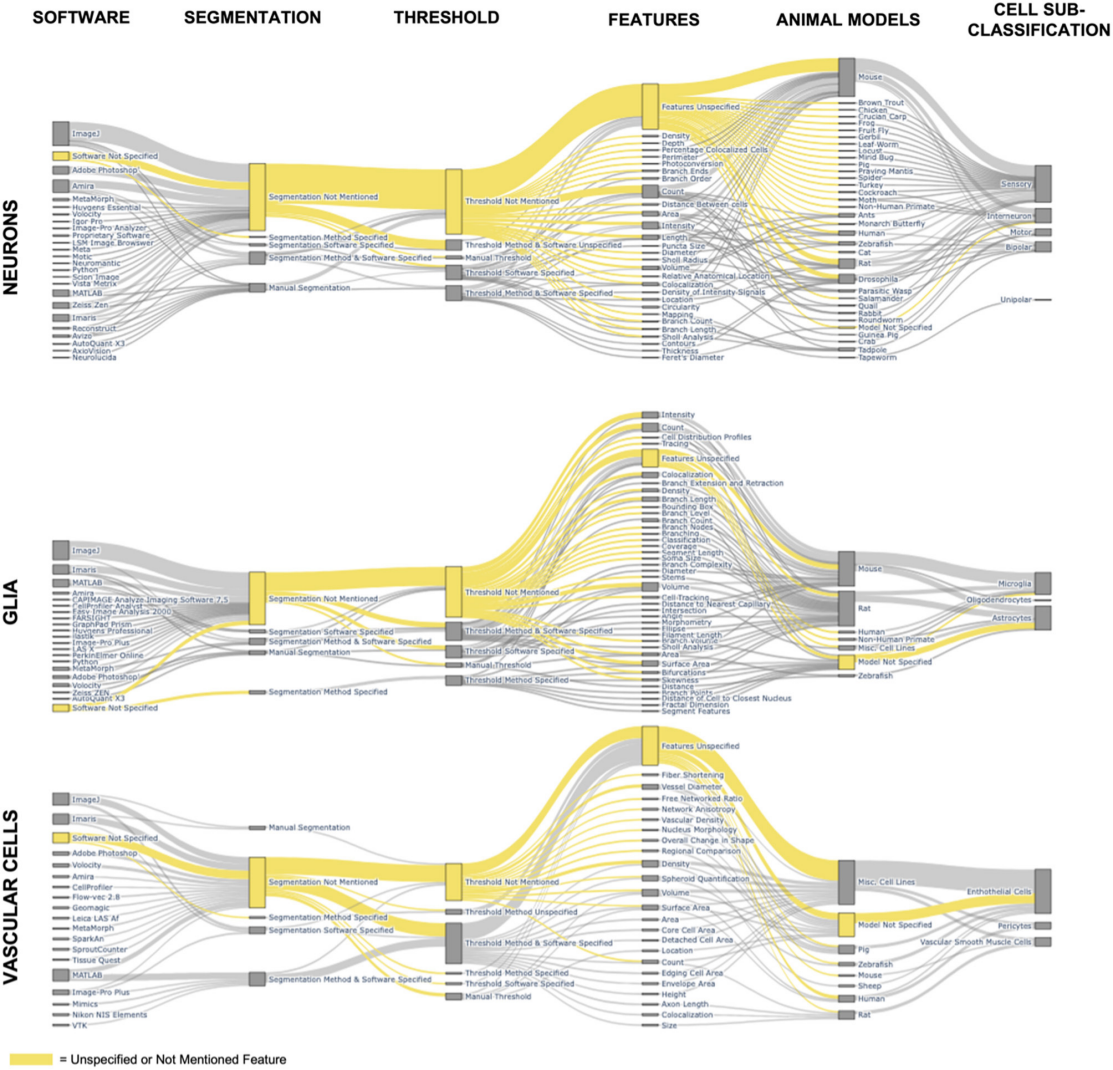
Feature combinations in published immunofluorescent cell papers. The Sankey diagrams depict the combination of software, image processing methods, quantified cell features, animal model, and cell sub-group classification for all major cell groups of the CNS for all papers using confocal microscopy. The node height, boxes corresponding to a specific feature, is determined by the number of papers mentioning that feature. The edge width, connecting line between two nodes, is determined by the number of papers that include the features from each of the connected nodes. The yellow nodes and edges represent all papers that leave information unspecified or unmentioned.

The most basic image processing step for fluorescent cell images is a brightness, or contrast, adjustment, which involves shifting the upper and lower bounds of lightness in an image. Brightness adjustments are most commonly applied whenever a fluorescent image transfers from the acquisition software to visualization or processing software. Beyond brightness adjustments, other image processing techniques are common for quantifying cell features such as cell count, area, or branching features. The more advanced image processing techniques can include multiple algorithms with tens of unique variable inputs that are not reproducible without sufficient methodology described in a publication. Since brightness adjustments are so common, the prevalence of immunofluorescent-image containing papers mentioning image processing should be similar to the prevalence of publications that mention CNS cells and fluorescent microscopy. Yet, as we demonstrated in our analysis of 9,000 CNS papers, there is a 10-fold decrease in papers that describe image processing methods. Therefore, we sought to further understand image processing publication habits by conducting a deep dive into the papers published with confocal microscopy, the most common type of fluorescent microscopy (Figure 3).

### 4.1. Commonly published experimental and cell features in image processing of CNS cells

The defined set of experimental features are animal models used, features quantified, image processing methods of thresholding and segmentation, and software used. We included animal models to show the wide application of fluorescent cell imaging from cell lines to humans. Quantified cell features such as count, area, or branch/process features were tagged and included as they provided a reference to common analyses. We included thresholding and segmentation methods to gain insight into how advanced image processing methods were included within publications. Finally, we included the software used to show the wide variety of platforms and coding languages used in image processing. The variety of both software and coding languages complicates image processing reproducibility by introducing additional variables while, conversely, offering more resources for researchers to analyze fluorescent cell images.

Animal models are more common than cell line models as the source for imaging with confocal microscopy, with mice as the most common study animal returned in our search, followed by rats. This is not surprising, seeing that rodent models are one of the most common research specimens (Bryda, 2013). The Sankey visualization (Figure 3) shows that sensory receptor cells are studied the most widely across animal species ranging from insects to fish and mammals. Meanwhile, endothelial cells are the only CNS cell more commonly studied in cell lines than in animal models in the 9,000 papers analyzed in this search.

By looking at the node sizes of quantified cell features, the most common cell features published are count and intensity. It is likely that count and intensity are the most easily accessed features by researchers without requiring extensive image processing procedures. Another consideration is that count can be completed manually without automated processing, although automation significantly increases throughput. The papers with robust feature quantification show the possibility of increased cell quantification from fluorescent images that could be applied to many of the other papers cited. However, there are several barriers that are keeping fluorescent imaging from “leveling” up to the more in-depth analysis of fluorescent images, which include: (1) expertise needed for high-quality imaging, (2) expertise needed for robust and unbiased image processing, (3) software support for consistent and detailed image processing methodologies, (4) lack of reporting on specific variables for doing quantification, (5) thresholding and segmentation barriers described in the Section 4.2, and (6) interpretation of the quantified features that maintain scientific relevance without bias. Previous literature and our review emphasize that with more robust and detailed image processing reporting, there is potentially limitless possibility to increase the amount of data gained from immunofluorescent images of CNS cells.

### 4.2. Thresholding and segmentation underuse for quantifying CNS cells

Thresholding and segmentation enable enhanced cell quantification techniques. While a lot can be gleaned from fluorescent images of cells based on qualitative analysis of images, every image of a cell holds a wealth of information beyond the precursory glance. All quantified features from the literature search containing the MeSH terms “Microscopy, Confocal” AND “Image Processing, Computer-Assisted” are included in Figure 3 and Supplementary Table 3. The most prevalent quantified feature was cell count, followed by volume, then surface area, then length, intensity, colocalization, and cell density. Volume is used specifically with 3D rendering from confocal z-stacks to measure the entire fluorescent volume of a cell. Meanwhile, length can be ascribed to the entire length of a single cell, subcellular features such as branching, or the length of the soma. Intensity and colocalizations are stain dependent. Intensity measures the signal intensities of the stain, and colocalization measures the amount of overlap between two stains of distinct color channels. An important note for Figure 3 is that a few papers often would add a significant number of quantified features to the list of features for that specific cell type due to their own extensive analysis.

Thresholding for cell researchers is an incredibly important and undervalued skill. Thresholding is the simplest method of segmenting cells. Applying a threshold to a cell image is the process of converting an image from grayscale to binary. The two major groups of thresholds of relevance to this review are manual thresholds and algorithmic thresholds. Manual thresholding is the process of an individual user—often with high skill and expertise in a specific cell type's biology—adjusting the pixel cutoff for binarization to produce the user's interpretation of the “highest quality” image. Manual thresholds can introduce significant bias into samples and, without proper reporting of threshold values, cause difficulties in reproducibility. Algorithmic thresholds aim to reduce bias while improving image segmentation and thresholding outcomes by using different mathematical and statistical models. It is worth noting that even algorithmic thresholds can have a bias based on the variable inputs used, the algorithm chosen, and changes in inputs and algorithms across experimental groups. With proper reporting, the algorithmic method should be less biased if applied identically across groups than manual thresholding and therefore algorithm-based thresholding is a continually growing area of interest for high throughput image processing.

Beyond thresholding as the simplest method to segment cells, other segmentation methods include machine learning-based segmentation, manual segmentation, and watershed-based segmentation. Many researchers currently focus on improving automatic segmentation methods for cell analysis, including automated 3D cell detection in human-derived cardiospheres (Salvi et al., 2019), nucleus-informed convolutional neural networks for eukaryotic cell segmentation (Korfhage et al., 2020), and automated watershed segmentation for cell nuclei annotation (Englbrecht et al., 2021) as just a few examples among many others. Automatic segmentation methods are necessary for high-throughput and batch processing of large experiments with hundreds to thousands of cell images. Batch processing and high-throughput analysis are limited by a lack of easily applied methods, the time component of accurate segmentation, and quantifiable characteristics to define how well a segmentation took place. Thresholding and cell segmentation are major bottlenecks in advanced cell quantification.

Both the threshold methodology and segmentation methodology sections of our Sankey diagrams emphasize both the underuse of these methods and the lack of published information for reproducing results (Figure 3). The yellow coloring highlights that across all types of cells, most papers in our search do not report either segmentation or thresholding. Of those that do use a thresholding or segmentation method, most of the publishing details are unmentioned; often, only the software or only the method is mentioned without enough detail included in the paper to reproduce the results. Supplementary Table 3 provides further information on which papers in our search used each method to quantify specific cell features. Overall, not all images have to undergo segmentation for quantification. Although thresholding is a more common image-processing practice than segmentation, the highest publication count, including the term “threshold,” is 10. Even further, only two of the ten publications were returned if the MeSH term “Image Processing, Computer-Assisted” was included in the search, suggesting thresholding is not considered image processing to either PubMed or the authors.

Covering all the different thresholding methods is not within the scope of this review. For a more detailed guide, we refer the reader to Aaron and Chew (2021) publication on biological image processing workflows. For the field to overcome the threshold and cell segmentation bottleneck, the authors recommend a needed improvement in cell image processing publishing documentation and standards. Based on the results from our systematic review, improvements include requiring standard publication methodologies for common image processing techniques, consistently tagging publications using thresholding with the MeSH term “Image Processing, Computer-Assisted,” and providing enough image processing detail for an outside researcher to reproduce images and results accurately. An extensive review of fluorescent microscopy methods reporting was published by Montero Llopis et al. (2021). Our recommendation is supported by the fact that thresholding methods have been reviewed in past articles, including a 1989 assessment of automated thresholds (Sieracki et al., 1989) and Healy et al. (2018) threshold-based segmentation comparison in glial cells. In Section 3, we see that while these reviews contribute important knowledge to the field, they have not made a significant impact on changing image processing practices. Increased adaption of image processing methods can likely only occur alongside improved methodology reporting.

Even if a researcher can easily access another paper with a CNS cell type of interest, researchers will still have difficulty following the methodology for a quantification method if the software is inaccessible. Deciding on the “best” image processing software for an immunofluorescent cell quantification task can be a complicated decision. Many software programs are available, as demonstrated by the 52 outlined in Table 1 and Supplementary Table 4, and many of the available software programs have similar capabilities. Some other reviews already exist that compare and discuss software for fluorescent cell image processing, such as a review of free software tools from Hamilton (2009) and Wiesmann et al. (2015) review of quantification in fluorescent microscopy images. Often, the “best” software to use is that which is the most accessible and best fits the expertise of the authors. In the next section, we cover topics related to software choices, such as accessibility and necessary expertise.

### 4.3. Determining the “best” software for image processing CNS cells

The most popular software platform for immunofluorescent cell image processing is ImageJ—we lumped together both the basic software and the FIJI implementation. ImageJ is supported by the National Institutes of Health and is an open-source, lightweight software that can be downloaded on most computers and laptops with a friendly graphical user interface (Schindelin et al., 2012; Schneider et al., 2012). The open-source nature and popularity of ImageJ contribute to an infrastructure of tutorials: a simple Google search of “YouTube “ImageJ”” returns 973,000 results, and “YouTube “ImageJ Tutorial”” returns 3,840. Price and available training are likely contributing factors to ImageJ's overall popularity. The second most popular software is Imaris, which has a hefty price tag of at least $13,000 for the base software and $45,000 for a single license for the cell segmentation packages used in many of the papers cited in this review. Beyond the original license purchase, Imaris—and many other software programs—may require regular maintenance fees, as well. Imaris requires an expensive computer with solid-state drives and multiple graphical processing units (GPUs). It is a large economic burden for a single lab to purchase and maintain the Imaris license. Therefore, the most common way to access software like Imaris is through an imaging core facility. The benefits of such a high price tag are an enhanced graphical user interface (GUI) and speedy processing with the purchase of the necessary supporting computer infrastructure, often accompanied by personalized tech support for specific data and analysis needs.

For labs with coding or data science expertise, another option is to use a coding language for personalized algorithm deployment. Researchers have the most control over the relevant variables in their analysis if they can write their own code. From our review, MATLAB and Python are the most cited coding languages in CNS cell publications. For both languages, many open-source packages exist for different aspects of image processing. Unlike Python, MATLAB does have a cost associated with its use, although the cost of MATLAB is significantly lower than Imaris and even lower with a student or academic license. Meanwhile, Python a has a similar advantage to ImageJ in that it is free, tutorials are abundant, and most code is open-source. The downside to using a coding language to write or access code is that it does take a higher amount of skill level and can take more time than a well-written GUI. Well-written packages with GUIs built-in Python like CellProfiler do exist, which integrate a cell-specific software with a build-it-yourself solution. In general, the scientists analyzing cells will have the best understanding of what expertise is on the research team and which options are available. If CNS researchers with expertise in immunofluorescent cell imaging become more frequent users of advanced image processing techniques for cell analysis, then new data and analysis can enable further understanding of CNS cells and their role in physiology, health, and disease.

## 5. Conclusion and future directions

Image processing is key to increasing insight from the thousands of CNS cell papers that already use immunofluorescent imaging. However, our systematic review showed that < 10% of papers with immunofluorescent images include image processing in their methods. We showed that image processing of immunofluorescent CNS cell images is underreported. Our deeper analysis of the wide variety of models, features, software, and methods used for imaging CNS cells provides an assessment of current practices in image processing and also a resource to find gaps in areas of application of image processing, and in areas for improvement within cell analyses and microscopy practices. Our assessment documented the most common cell analysis software and their quantification capabilities, creating a reference to CNS cell researchers to identify cell features and the most effective image processing methods for cell feature analysis. We also graphically represented the relationship between different microscopy techniques to highlight opportunities for future experiments combining microscopy methods to capture unique aspects of CNS cells. Given the disconnect between the prevalence of immunofluorescent imaging of CNS cells and the reporting of imaging processing, we conclude with a guide for improving image processing (Box 1). By implementing improved image processing practices in current research and publishing detailed and rigorous imaging processing methods, the CNS cell research field can increase the impact and quantitative data outputs of immunofluorescent CNS cell images.

Box 1 **Roadmap to enhancing cell quantification with improved image processing.**

Image processing enhances the analysis capabilities of immunofluorescent cell images. The five simple steps below are a guide to getting more out of cell images with image processing.

**Step 1: Select a stain and fluorescent microscopy method**

- Store images in the highest quality image format and preserve metadata from acquisition—preferably use a lossless file format such as the equipment specific file format, PNG, or TIFF files rather than JPG, which compresses the file. Rigano et al. (2021) and Ropelewski et al. (2022) provide reviewers of metadata specifications.
- Determine a file naming and storing structure before acquisition to make analysis easier and refrain from using spaces in file names.

**Step 2: Determine features to quantify**

- Figure 3 provides a reference to features that can be quantified for different CNS cell types.
- Ensure the desired feature is accurately captured by the selected staining and microscopy method.

**Step 3: Select a software**

- Table 1 provides an overview of the software, including cost, function, and previous use in the literature.
  ° Assess the amount of time available to learn a new software.
  ° Assess software and training available in imaging cores at your university or research institute.
- Confirm the selected software can quantify your features of interest.

**Step 4: Select thresholding or segmentation methods**

- Begin with the simplest method offered by your selected software and work toward a more advanced method as your expertise and application matures.
- Save intermediate images of all major steps for visual reference that the algorithm is working the way you intend.
- Select one algorithm and set of inputs for your entire experimental set to reduce bias and image processing variation.

**Step 5: Record and publish methodological variables**

- Record software information, including the version and distribution.
- Record every input to the software, from opening the image to the final results.

## Data availability statement

The datasets presented in this study can be found in online repositories. The names of the repository/repositories and accession number(s) can be found below: Zenodo, https://doi.org/10.5281/zenodo.7651627.

## Author contributions

Conceptualization, project administration, and writing—review and editing: HH and EN. Methodology, formal analysis, and visualization: HH and T-JL. Software, validation, and resources: HH. Investigation, data curation, and writing—original draft: HH, T-JL, SJ, and KD. Supervision and funding acquisition: EN. All authors contributed to the article and approved the submitted version.
