# Supplementary material for: Prevalence and practices of immunofluorescent cell image processing: a systematic review
Source: Front Cell Neurosci. 2023 Jul 20;17:1188858. doi: 10.3389/fncel.2023.1188858 (PMC10400723; doi:10.3389/fncel.2023.1188858)
Supplement: Supplementary file 1 [file Table_1.DOCX]

Supplementary Material

Prevalence and Practices of Immunofluorescent Cell Image Processing a Systematic Review

Hawley Helmbrecht^1^, Teng-Jui Lin^1^, Sanjana Janakiraman^2^, Kaleb Decker^1^, Elizabeth Nance^1,3^

^1^Department of Chemical Engineering, University of Washington, Seattle, Washington, USA

^2^Paul G. Allen School of Computer Science & Engineering, Seattle, Washington, USA

^3^Department of Bioengineering, University of Washington, Seattle, Washington, USA

*** Correspondence:**Elizabeth Nance
eanance@uw.edu

# Supplementary Figures and Tables

For more information on Supplementary Material and for details on the different file types accepted, please see [here](https://www.frontiersin.org/guidelines/author-guidelines#supplementary-material).

## Supplementary Figures


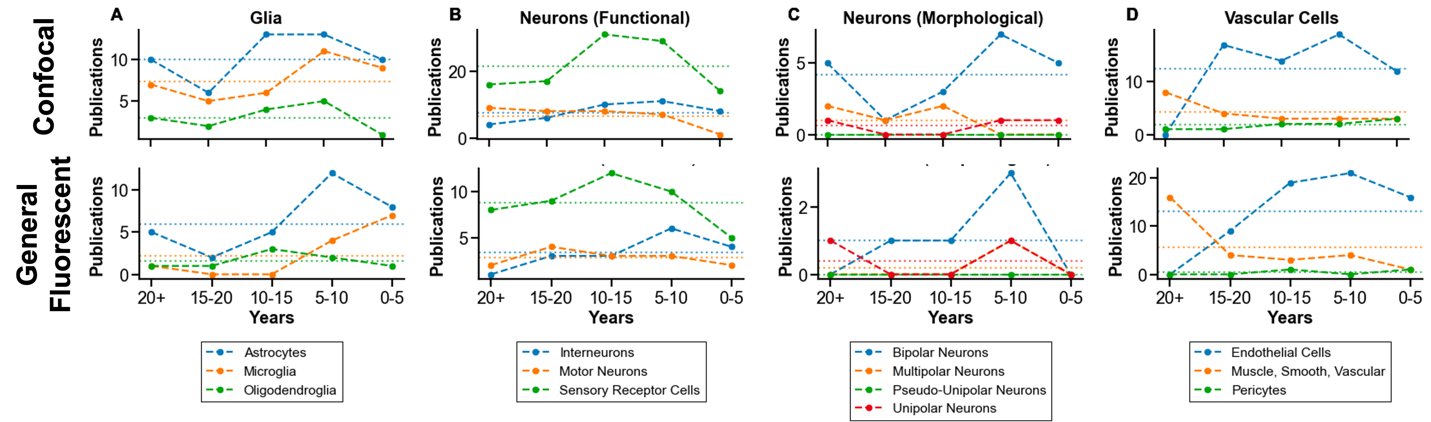


**Supplementary Fig 1.** The publication count over time of papers using confocal and general fluorescent microscopy with image processing. Each column represents a different cell classification group A) Glia, B) Neurons (functional classifications), C) Neurons (morphological classifications), and D) Vascular Cells. The key for each columns sub-group classifications is included below the column.

**1.2 Supplementary Tables**

| **Supplementary Table 1: Number of publications for each cell group and fluorescent microscopy technique** | | | | | | | | | |
| --- | --- | --- | --- | --- | --- | --- | --- | --- | --- |
| **Cell Group** | **Cell Type** | **Confocal, "Microscopy, Confocal"[MeSH]** | | **"Microscopy, Fluorescence"[MeSH] AND “General Fluorescent Microscopy”** | **Fluorescent Widefield, "Microscopy, Fluorescence"[MeSH] AND "Widefield"** | **Multiphoton, "Microscopy, Fluorescence, Multiphoton"[MeSH]** | **"Microscopy, Fluorescence"[MeSH] AND "Light Sheet"** | **"Microscopy, Fluorescence"[MeSH] AND "Total Internal Reflection"** | **"Microscopy, Fluorescence"[MeSH] AND "Super Resolution"** |
| **Neurons** | **Functional Differences** | | | | | | | | |
|  | Motor ("Motor Neurons"[MeSH]) | 250 | 242 | | 0 | 5 | 2 | 0 | 1 |
|  | Sensory ("Sensory Receptor Cells"[MeSH]) | 1091 | 899 | | 4 | 30 | 2 | 2 | 4 |
|  | Interneuron ("Interneurons"[MeSH]) | 342 | 177 | | 3 | 17 | 3 | 2 | 0 |
|  | **Morphological Differences, "Neurons"[MeSH] AND x** | | | | | | | | |
|  | Multipolar | 28 | 27 | | 0 | 0 | 0 | 0 | 0 |
|  | Unipolar | 12 | 11 | | 0 | 0 | 0 | 0 | 0 |
|  | Bipolar | 198 | 102 | | 2 | 7 | 0 | 2 | 0 |
|  | Pseudo-unipolar | 0 | 1 | | 0 | 0 | 0 | 0 | 0 |
| **Glia** | Microglia ("Microglia"[MeSH]) | 324 | 182 | | 2 | 37 | 1 | 0 | 1 |
|  | Astrocytes ("Astrocytes"[MeSH]) | 588 | 492 | | 7 | 54 | 3 | 11 | 6 |
|  | Oligodendrocytes ("Oligodendroglia"[MeSH]) | 219 | 214 | | 0 | 15 | 0 | 0 | 0 |
|  | Ependymal Cells ("Ependymoglial Cells" [MeSH]) | 22 | 13 | | 0 | 0 | 0 | 0 | 0 |
|  | Endothelial Cells ("Endothelial Cells"[MeSH]) | 962 | 1,173 | | 8 | 38 | 8 | 15 | 22 |
|  | Pericytes ("Pericytes"[MeSH]) | 82 | 63 | | 1 | 8 | 0 | 0 | 0 |
|  | Vascular Smooth Muscle Cells ("Muscle, Smooth, Vascular"[MeSH]) | 410 | 420 | | 1 | 8 | 0 | 4 | 0 |
| **Total** | | **4696** | **4186** | | **29** | **221** | **19** | **36** | **34** |

| **Supplementary Table 2: Publication count for each cell type including different image processing search terms** | | | | | | | | | | | |
| --- | --- | --- | --- | --- | --- | --- | --- | --- | --- | --- | --- |
|  | | **Number of Publications - Adding in the Search Term: ("Image Processing, Computer-Assisted"[MeSH])** | | **Number of Publications Using Threshold or Segment AND ("Image Processing, Computer-Assisted"[MeSH])** | | | | **Number of Publications Using Threshold or Segment AND image AND x** | | | |
| **Cell Group** | **Cell Type** | **Confocal*, "Microscopy, Confocal"[MeSH]** | **"Microscopy, Fluorescence"[MeSH] AND “General Fluorescent Microscopy”** | **Confocal*, "Microscopy, Confocal"[MeSH]** | | **General Fluorescent Microscopy, X AND "Microscopy, Fluorescence"[MeSH]** | | **Confocal*, "Microscopy, Confocal"[MeSH]** | | **General Fluorescent Microscopy, X AND "Microscopy, Fluorescence"[MeSH]** | |
|  | |  | | **Threshold** | **Segment** | **Threshold** | **Segment** | **Threshold** | **Segment** | **Threshold** | **Segment** |
| **Neurons** | **Functional Differences** | | | | | | | | | | |
|  | Motor ("Motor Neurons"[MeSH]) | 33 | 14 | 3 | 2 | 1 | 0 | 3 | 6 | 1 | 0 |
|  | Sensory ("Sensory Receptor Cells"[MeSH]) | 106 | 44 | 2 | 11 | 2 | 3 | 10 | 34 | 5 | 15 |
|  | Interneuron ("Interneurons"[MeSH]) | 37 | 17 | 1 | 3 | 0 | 3 | 2 | 7 | 3 | 5 |
|  | ****Morphological Differences, "Neurons"[MeSH] AND x** | | | | | | | | | | |
|  | “Multipolar” | 5 | 1 | 0 | 1 | 0 | 0 | 0 | 1 | 0 | 0 |
|  | “Unipolar” | 3 | 2 | 0 | 0 | 0 | 1 | 0 | 0 | 0 | 0 |
|  | “Bipolar” | 21 | 5 | 0 | 2 | 0 | 0 | 1 | 6 | 1 | 2 |
|  | “Pseudo-unipolar” | 0 | 0 | 0 | 0 | 0 | 0 | 0 | 0 | 0 | 0 |
| **Glia** | Microglia ("Microglia"[MeSH]) | 36 | 10 | 1 | 4 | 0 | 0 | 4 | 7 | 1 | 1 |
|  | Astrocytes ("Astrocytes"[MeSH]) | 49 | 30 | 3 | 3 | 0 | 1 | 3 | 3 | 2 | 2 |
|  | Oligodendrocytes ("Oligodendroglia"[MeSH]) | 15 | 8 | 1 | 1 | 0 | 0 | 1 | 3 | 0 | 1 |
|  | Ependymal Cells ("Ependymoglial Cells" [MeSH]) | 1 | 0 | 0 | 0 | 0 | 0 | 0 | 1 | 0 | 1 |
| **Vascular Cells** | Endothelial Cells ("Endothelial Cells"[MeSH]) | 60 | 64 | 0 | 2 | 1 | 3 | 1 | 8 | 1 | 12 |
|  | Pericytes ("Pericytes"[MeSH]) | 9 | 2 | 0 | 0 | 0 | 0 | 2 | 1 | 0 | 1 |
|  | Vascular Smooth Muscle Cells ("Muscle, Smooth, Vascular"[MeSH]) | 21 | 28 | 1 | 3 | 2 | 3 | 1 | 6 | 2 | 7 |
| **Total** | | 412 | 233 | 12 | 34 | 6 | 14 | 29 | 89 | 16 | 50 |

| **Supplementary Table 3: Models, Features, Techniques, and Software used for Publications using Confocal Microscopy and Image Processing since 2010** | | | | | | |
| --- | --- | --- | --- | --- | --- | --- |
| **Cell Group** | **Cell Type** | **Models** | **Quantified Features** | **Threshold Techniques Used** | **Segmentation Techniques Used** | **General Image Processing Software Used** |
| Neurons | **Functional Differences** | | | | | |
|  | **Motor** | Crab [1], Drosophila [2], Guinea Pig [3], Mouse [4, 5, 6, 7, 8, 9] | Area [9], Count [5, 8], Feret's Diameter [10], Density [9], Density of Intensity Signals [6], Intensity [3, 4, 6], Perimeter [9], Photoconversion [5], Length [2], Relative Anatomical Location [1], Sholl Radius [2], Volume [6] | Grow and Shrink Volumes of Interest [6], Threshold to Lowest Background Signal [10] |  | Amira [2], ImageJ [3, 4, 7, 8, 10], MATLAB [6 - link to software in paper], MetaMorph [5], VistaMetrix [1], Zeiss ZEN [1] |
|  | **Sensory** | Ants [11, 12, 13], Brown Trout [14], Chicken [15], Cockroach [16], Crucian Carp [14], Drosophila [17, 18, 19, 20, 21], Frog [22], Fruit Fly [23], Gerbil [24], Human [25, 26], Leaf Worm [27], Locust [28], Mirid Bug [29],Moth [31], Monarch Butterfly [30], Mouse [24, 32, 33, 34, 35, 36, 37, 38, 39, 40, 41, 42, 43, 44, 45], Parasitic Wasp [46], Pig [15], Praying Mantis [47], Quail [48], Rat [37, 49], Roundworm [50], Spider [51], Turkey [15], Zebrafish [52, 53, 54] | Area [32], Branch Ends [44], Branch Order [44], Colocalization [48], Contours [19], Density [32], Depth [32], Distance Between Cells [44], Intensity [19, 34, 42], Length [11, 44, 46], Location [19], Percentage Colocalized Cells [42], Thickness [53], Volume [11, 30, 46, 50] | Contour Based Thresholds [19], Histogram Based Threshold [28], Imaris Based - Method Unspecified [41, 44], Intensity Threshold [54 - Link to Software Available], Log-Linear Threshold [48], Manual [40], Mean of Two Brightness Values [53] | Amira - Based - Brush and Interpolate [29], Amira Based - Manual Segmentation [22], Amira Based - Method Unspecified [13, 23], Histogram-Based Threshold for Segmentation [28], Imaris Based - Manual Segmentation [41, 44], Imaris Based - Surfaces Segmentation [43], Intensity Threshold [42], K-Means Based Segmentation [42], Manual [16, 42], Threshold Based Segmentation [27, 40, 45] | Adobe Photoshop [12, 18, 24, 35, 49, 52], Amira [11, 12, 13, 20, 21, 22, 23, 28, 29, 30, 31, 51], AutoQuant X3 [44], Avizo [16, 27, 47], Huygens Essential [33], Igor Pro [34], ImageJ [18, 21, 27, 28, 30, 33, 34, 40, 42, 46, 48, 50, 51, 52], Image-Pro Analyzer [48], Imaris [25, 38, 41, 43, 44], LSM Image Browser [36], MATLAB [19, 40, 41, 53, 54], Motic [48], Meta [48], Neurolucida [44], Python [40], Scion Image [32], Volocity [49], Zeiss ZEN [26, 24, 42, 51], Proprietary Software [37] |
|  | **Interneuron** | Cockroach [55], Drosophila [56], Moth [57], Mouse [58, 59, 60, 61, 62, 63, 64, 69], Non-Human Primate [65], Rabbit [61], Rat [59, 61, 66, 67, 68] | Area [61], Circularity [59], Colocalization [59, 62], Count [60, 61, 62], Intensity [59, 60], Puncta Size [59] | ImageJ Based - Method Unspecified [60], Manual Threshold [61], Method Unspecified [55, 59], Otsu Threshold [69], Size Based Threshold [65] | Deep Neural Network Based Segmentation [69], Manual Segmentation [65, 70], Method Unspecified [60], Threshold-Based Segmentation [59, 61] | Amira [55], AxioVision [70], ImageJ [56, 59, 60, 61, 67, 68, 69, 70], Imaris [61, 68, 69], MATLAB [69, 70], MetaMorph [65], Reconstruct [58, 65], Volocity [66], Zeiss ZEN [69] |
|  | **Morphological Differences** | | | | | |
|  | **Multipolar** | --- | --- | --- | --- | --- |
|  | **Unipolar** | Tapeworm [71] | Mapping [71] |  |  | Adobe Photoshop [71], Amira [71] |
|  | **Bipolar** | Cat [72, 73], Drosophila [74], Human [75, 76], Mouse [72, 73], Rat [72, 73, 77], Salamander [37], Tadpole [78], Zebrafish [79] | Area [75], Branch Length [78], Branch Count [78], Count [75, 78, 79], Diameter [75], Length [79], Location [79], Sholl Analysis [78] | Intensity Threshold [72], Manual Threshold [76], Neuromantic Based - Method Unspecified [78], Otsu Threshold [77], Size Based Threshold [72] | Threshold-Based Segmentation [77] | Adobe Photoshop [72, 74, 76], Amira [77, 79], ImageJ [74, 75, 76, 78], Imaris [79], MetaMorph [79], Neuromantic [78], Zeiss ZEN [74], proprietary software [37] |
|  | **Pseudo-unipolar** | --- | --- | --- | --- | --- |
| **Glia** | **Microglia** | Human [80, 81], Mouse [81, 82, 83, 84, 85, 86, 87, 88, 89, 90, 91], Misc Cell Line [92], Non-Human Primate [93], Rat [94, 95, 96, 97] | Area [89, 96], Angle [96], Branch Count [97], Count [82, 87, 88, 89, 96], Colocalization [87, 94], Diameter [82], Density [82, 89], Ellipse [96], Distance to Nearist Capillary [88], Filament Length [97], Intensity [83, 84, 86, 89], Intersection [91], Process Length [84], Process Extention and Retraction [84] , Process Count [84], Process Complexity [88], Sholl Analysis [97], Surface Area [97], Volume [88, 89, 97] | Hierarchical Local Threshold Method [98], ImageJ Based - Method Unspecified [82], Imaris Based - Local Contrast Threshold [97], Intensity Threshold [84], Manual Threshold [90], Minimum Volume Threshold [81], MATLAB Based - Method Unspecified [88], Otsu Threshold [96] | Imaris Based - Filament Tracer Manual Segmentation [93], MATLAB Based - Method Unspecified [85], Photoshop Based - Manual Segmentation [80], Supervised Segmentation via Threshold and Dilation [81], Threshold Based Segmentation [96, 98 - link to code available in paper] | Adobe Photoshop [80, 85], Amira [85], CAPIMAGE Analyze Imaging Software 7.5 [89], ImageJ [81, 82, 83, 84, 85, 86, 90, 91, 92, 93], ilastik [81], Imaris [87, 89, 90, 91, 93, 97], LAS X [92], MATLAB [81, 83, 85, 88, 96], MetaMorph [95], GraphPad Prism [86], Volocity [89], Zeiss ZEN [81] |
|  | **Astrocytes** | Mouse [99, 100, 101, 102, 103, 104, 105], Misc Cell Lines [104, 106, 107], Non-Human Primate [108], Rats [100, 107, 109, 110, 111, 112], Zebrafish [113] | Area [113], Bifurcations [112, 114], Bounding Box [112], Branching [112], Branch Length [110, 115], Branch Points [114], Branch Volume [115], Cell Tracking [107], Cell Distribution Profiles [105], Colocalization [99, 106, 109], Count [102, 103, 115], Coverage [110, 113], Classification [112], Density [110], Distance [113], Distance of Cell to Closest Nucleus [114], Fractal Dimension [114], Intensity [110, 115], Leaf Nodes [112], Leaf Length [112], Leaf Level [112], Morphometry [101], Segments [114], Segment Length [112], Skewness [112 ,114], Soma Size [112], Stems [112, 114], Surface Area [110, 112, 114, 115], Tracing [103] | Cell Analyst Based - Method Unspecified [101], Automated Coverage Method [114], ImageJ - Based Threshold Based on "All Filled" Cells [100], Imaris-Based Intensity Threshold [99], Intensity Based Threshold [104], Manual Intensity Threshold [109, 113], MATLAB-Based Method Unspecified [116], Mean Intensity Based Threshold [117] | ImageJ Neurite Tracker Based - Method Unspecified [100], Imaris Based - Manual Segmentation [109], Imaris Based Segmentation - Method Unspecified [105], Machine Learning Based Segmentation [114], Manual Segmentation [115], Method Unspecified [112], Watershed Based Segmentation [104] | Adobe Photoshop [106], Amira [113], AutoQuant X3 [109], CellProfiler Analyst [101], Easy Image Analysis 2000 [110], FARSIGHT [112], Huygens Professional [117], ImageJ [100, 102, 103, 104, 105, 106, 107, 117], Image-Pro Plus [99], Imaris [99, 102, 109], MATLAB [116, 118], MetaMorph [104, 113], PerkinElmer Online [110], Python [118], Volocity [106, 115] |
|  | **Oligodendrocytes** | Mice [119] | Intensity [119] |  |  | ImageJ [119] |
| **Vascular Cells** | **Endothelial Cells** | Human [120], Misc Cell Lines [121, 122, 123, 124, 125, 126, 127, 128, 129, 130, 131, 132, 133, 134], Sheep [135], Pig [133, 136], Zebrafish [129, 137] | Area [130], Core Cell Area [130], Count [138], Density [130, 135, 138], Detached Cell Area [130], Edging Cell Area [130], Envelope Area [130], Fiber Shortening [123], Free Networked Ratio [139], Height [122], Location [140], Overall Change in Shape [136], Network Anisotropy [139], Nucleus Morphology [136], Regional Comparison [136], Spheroid Quantification [129, 130], Surface Area [120, 122 140], Vascular Density [139], Vessel Diameter [136, 138, 139], Volume [120, 122, 130, 140] | Amira Based - Method Unspecified [121], Background Intensity Based Threshold [126], Contrast Enhancement Threshold [133], ImageJ Based - Manual Threshold [140], Imaris - Absolute Intensity Threshold [141], Intensity Based Threshold [127, 132], Maximum Threshold [122], Minimum Threshold [122], Otsu Threshold [131, 134], Photoshop Based Manual Threshold [142], Unspecified High Contrast Thresohld [130], Unspecified Mathematical Thresholding [128], Unspecified Threshold Method [143] | Fiberscore Algorithm [128], K-Means Clustering Segmentation [131], Manual [120], Method Unspecified [133, 136], Mimics Based - Manual Segmentation [122], Threshold Based Segmentation [127, 130, 132] | Adobe Photoshop [141, 142], Amira [121], Flow-vec 2.8 [124], Geomagic [122], Image-Pro Plus [127, 136, 143], ImageJ [120, 121, 129, 133, 136, 138, 140], Imaris [120, 124, 133, 138, 141, 142], MATLAB [130, 131, 132, 139], Mimics [122], Nikon NIS Elements [132], SproutCounter [126], TissueQuest [123], Volocity [123, 125], VTK [122] |
|  | **Pericytes** | Human [144], Mouse [145], Misc Cell Lines [134, 146] | Density [146] | Intensity Based Threshold [144, 146], Otsu Threshold [134], Size Based Threshold [146] | K-Means Clustering Based Segmentation [146] | Leica LAS AF [144], MATLAB [134, 146] |
|  | **Vascular Smooth Muscle Cells** | Mouse [147], Rat [148, 149], Misc Cell Lines [148, 150] | Axon Length [149], Colocalization [149], Count [149], Size [149] | Hysteresis Thresholding [149], Otsu Threshold [150], Wavelet Thresholding [148] |  | CellProfiler [150], MetaMorph [147], MATLAB [149], SparkAn [147] |

| **Supplementary Table 4. Other Mentioned Software with Only One Associated Publication** | |
| --- | --- |
| **Software Name** | **Developer/Parent Company** |
| AxioVision | Zeiss |
| CAPIMAGE Analyze Imaging Software 7.5 | -- |
| CellProfiler | Broad Institute |
| CellProfiler Analyst | Broad Institute |
| Easy Image Analysis 2000 | HORIBA |
| FARSIGHT | Roysam Laboratory |
| Flow-vec 2.8 | -- |
| Geomagic | 3D Systems |
| GraphPad Prism | GraphPad |
| Huygens Essential | Scientific Volume Imaging |
| Huygens Professional | Scientific Volume Imaging |
| Igor Pro | WaveMetrics |
| ilastik | ilastik |
| Image-Pro Analyzer | Media Cybernetics |
| LAS X | Leica |
| Leica LAS AF | Leica |
| LSM Image Browser | Zeiss |
| Meta | -- |
| Mimics | Materialise |
| Motic | Motic |
| Neurolucida | mbf Bioscience |
| Neuromantic | Darrn Myatt - University of Reading |
| Nikon NIS Elements | Nikon |
| PerkinElmer Online | PerkinElmer |
| Scion Image | -- |
| SparkAn | -- |
| SproutCounter | -- |
| TissueQuest | TissueGnostics |
| VistaMetrix | SkillCrest |
| VTK - The Visualization Toolkit | Kitware |

**Supplementary Table 3 References**

1 Swallie, S. E., Monti, A. M. & Blitz, D. M. Anatomical Organization of Multiple Modulatory Inputs in a Rhythmic Motor System. *PLOS ONE* 10, e0142956, doi:10.1371/journal.pone.0142956 (2015).

2 Vonhoff, F. & Duch, C. Tiling among stereotyped dendritic branches in an identified Drosophila motoneuron. *The Journal of Comparative Neurology* 518, 2169-2185, doi:10.1002/cne.22380 (2010).

3 Fernández-Alvarez, A., Gómez-Sena, L., Fabbiani, M. G., Budelli, R. & Abudara, V. Endogenous presynaptic nitric oxide supports an anterograde signaling in the central nervous system. *Journal of Neurochemistry* 118, 546-557, doi:10.1111/j.1471-4159.2011.07336.x (2011).

4 Tapia, C., Juan *et al.* Pervasive Synaptic Branch Removal in the Mammalian Neuromuscular System at Birth. *Neuron* 74, 816-829, doi:10.1016/j.neuron.2012.04.017 (2012).

5 Bolea, I., Gan, W.-B., Manfredi, G. & Magrané, J. in *Methods in Enzymology* 97-110 (Elsevier, 2014).

6 Fenrich, K. K., Zhao, E. Y., Wei, Y., Garg, A. & Rose, P. K. Isolating specific cell and tissue compartments from 3D images for quantitative regional distribution analysis using novel computer algorithms. *Journal of Neuroscience Methods* 226, 42-56, doi:10.1016/j.jneumeth.2014.01.011 (2014).

7 Gibbs, K. L., Kalmar, B., Sleigh, J. N., Greensmith, L. & Schiavo, G. In vivo imaging of axonal transport in murine motor and sensory neurons. *Journal of Neuroscience Methods* 257, 26-33, doi:10.1016/j.jneumeth.2015.09.018 (2016).

8 Žygelytė, E. *et al.* RetroDISCO: Clearing technique to improve quantification of retrograde labeled motor neurons of intact mouse spinal cords. *Journal of Neuroscience Methods* 271, 34-42, doi:10.1016/j.jneumeth.2016.05.017 (2016).

9 Dukkipati, S. S., Chihi, A., Wang, Y. & Elbasiouny, S. M. Experimental Design and Data Analysis Issues Contribute to Inconsistent Results of C-Bouton Changes in Amyotrophic Lateral Sclerosis. *eneuro* 4, ENEURO.0281-0216., doi:10.1523/eneuro.0281-16.2016 (2017).

10 Herron, L. R. & Miles, G. B. Gender-specific perturbations in modulatory inputs to motoneurons in a mouse model of amyotrophic lateral sclerosis. *Neuroscience* 226, 313-323, doi:10.1016/j.neuroscience.2012.09.031 (2012).

11 Kelber, C., Rössler, W. & Kleineidam, C. J. Phenotypic plasticity in number of glomeruli and sensory innervation of the antennal lobe in leaf-cutting ant workers (A. vollenweideri). *Developmental Neurobiology* 70, 222-234, doi:10.1002/dneu.20782 (2010).

12 Nakanishi, A., Nishino, H., Watanabe, H., Yokohari, F. & Nishikawa, M. Sex-specific antennal sensory system in the ant Camponotus japonicus: Glomerular organizations of antennal lobes. *The Journal of Comparative Neurology* 518, 2186-2201, doi:10.1002/cne.22326 (2010).

13 Kuebler, L. S., Kelber, C. & Kleineidam, C. J. Distinct antennal lobe phenotypes in the leaf-cutting ant (Atta vollenweideri). *The Journal of Comparative Neurology* 518, 352-365, doi:10.1002/cne.22217 (2010).

14 Døving, K. B., Hansson, K.-A., Backström, T. & Hamdani, E. H. Visualizing a set of olfactory sensory neurons responding to a bile salt. *Journal of Experimental Biology* 214, 80-87, doi:10.1242/jeb.046607 (2011).

15 Garlipp, M. A., Nowak, K. R. & Gonzalez-Fernandez, F. Cone outer segment extracellular matrix as binding domain for interphotoreceptor retinoid-binding protein. *The Journal of Comparative Neurology* 520, 756-769, doi:10.1002/cne.22773 (2012).

16 Watanabe, H., Haupt, S. S., Nishino, H., Nishikawa, M. & Yokohari, F. Sensillum-specific, topographic projection patterns of olfactory receptor neurons in the antennal lobe of the cockroach Periplaneta americana. *The Journal of Comparative Neurology* 520, 1687-1701, doi:10.1002/cne.23007 (2012).

17 Williamson, W. R. & Hiesinger, P. R. Preparation of Developing and Adult <em>Drosophila</em> Brains and Retinae for Live Imaging. *Journal of Visualized Experiments*, doi:10.3791/1936 (2010).

18 Pollarolo, G., Schulz, J. G., Munck, S. & Dotti, C. G. Cytokinesis remnants define first neuronal asymmetry in vivo. *Nature Neuroscience* 14, 1525-1533, doi:10.1038/nn.2976 (2011).

19 Sood, P., Johnston, R. J. & Kussell, E. Stochastic De-repression of Rhodopsins in Single Photoreceptors of the Fly Retina. *PLoS Computational Biology* 8, e1002357, doi:10.1371/journal.pcbi.1002357 (2012).

20 Domínguez-Cejudo, M. A. & Casares, F. Antero-posterior patterning of Drosophila ocelli requires an anti-repressor mechanism within the hh-pathway mediated by the Six3 gene Optix. *Development* 142, 2801-2809, doi:10.1242/dev.125179 (2015).

21 Rist, A. & Thum, A. S. A map of sensilla and neurons in the taste system ofdrosophilalarvae. *Journal of Comparative Neurology* 525, 3865-3889, doi:10.1002/cne.24308 (2017).

22 Elliott, K. L., Houston, D. W., Decook, R. & Fritzsch, B. Ear manipulations reveal a critical period for survival and dendritic development at the single-cell level in Mauthner neurons. *Developmental Neurobiology* 75, 1339-1351, doi:10.1002/dneu.22287 (2015).

23 Solari, P. *et al.* Morphological characterization of the antennal lobes in the Mediterranean fruit fly Ceratitis capitata. *Journal of Comparative Physiology A* 202, 131-146, doi:10.1007/s00359-015-1059-7 (2016).

24 Klotz, L. & Enz, R. MGluR7 is a presynaptic metabotropic glutamate receptor at ribbon synapses of inner hair cells. *The FASEB Journal* 35, doi:10.1096/fj.202100672r (2021).

25 Dauch, J. R., Lindblad, C. N., Hayes, J. M., Lentz, S. I. & Cheng, H. T. Three-dimensional Imaging of Nociceptive Intraepidermal Nerve Fibers in Human Skin Biopsies. *Journal of Visualized Experiments*, doi:10.3791/50331 (2013).

26 Talagas, M. *et al.* Intra‐epidermal nerve endings progress within keratinocyte cytoplasmic tunnels in normal human skin. *Experimental Dermatology* 29, 387-392, doi:10.1111/exd.14081 (2020).

27 Seada, M. A. & Ghaninia, M. Deep-tissue confocal imaging of the central projections of ovipositor sensory afferents in the Egyptian cotton leafworm, Spodoptera littoralis. *Micron* 82, 52-62, doi:10.1016/j.micron.2015.12.009 (2016).

28 Münch, D., Ott, S. R. & Pflüger, H.-J. Three-dimensional distribution of NO sources in a primary mechanosensory integration center in the locust and its implications for volume signaling. *The Journal of Comparative Neurology* 518, 2903-2916, doi:10.1002/cne.22396 (2010).

29 Xie, G.-Y. *et al.* Central Projection of Antennal Sensory Neurons in the Central Nervous System of the Mirid Bug Apolygus lucorum (Meyer-Dür). *PLOS ONE* 11, e0160161, doi:10.1371/journal.pone.0160161 (2016).

30 Heinze, S., Florman, J., Asokaraj, S., El Jundi, B. & Reppert, S. M. Anatomical basis of sun compass navigation II: The neuronal composition of the central complex of the monarch butterfly. *Journal of Comparative Neurology* 521, 267-298, doi:10.1002/cne.23214 (2013).

31 Zhemchuzhnikov, M. K., Pfuhl, G. & Berg, B. G. Tracing and 3-dimensional representation of the primary afferents from the moth ear. *Arthropod Structure & Development* 43, 231-241, doi:10.1016/j.asd.2014.04.001 (2014).

32 Ohtubo, Y. & Yoshii, K. Quantitative analysis of taste bud cell numbers in fungiform and soft palate taste buds of mice. *Brain Research* 1367, 13-21, doi:10.1016/j.brainres.2010.10.060 (2011).

33 Huang, L.-C. *et al.* Synaptic profiles during neurite extension, refinement and retraction in the developing cochlea. *Neural Development* 7, 38, doi:10.1186/1749-8104-7-38 (2012).

34 Hovis, K. R. *et al.* Activity Regulates Functional Connectivity from the Vomeronasal Organ to the Accessory Olfactory Bulb. *Journal of Neuroscience* 32, 7907-7916, doi:10.1523/jneurosci.2399-11.2012 (2012).

35 Bond, A. & Kinnamon, J. C. Microwave processing of gustatory tissues for immunohistochemistry. *Journal of Neuroscience Methods* 215, 132-138, doi:10.1016/j.jneumeth.2013.02.014 (2013).

36 Mulvaney, J. F. *et al.* Secreted Factor R-Spondin 2 is Involved in Refinement of Patterning of the Mammalian Cochlea. *Developmental Dynamics* 242, 179-188, doi:10.1002/dvdy.23908 (2013).

37 De Sevilla Müller, L. P., Liu, J., Solomon, A., Rodriguez, A. & Brecha, N. C. Expression of voltage-gated calcium channel α2δ4subunits in the mouse and rat retina. *Journal of Comparative Neurology* 521, 2486-2501, doi:10.1002/cne.23294 (2013).

38 Wright, G. D. & Horn, H. F. Three-dimensional image analysis of the mouse cochlea. *Differentiation* 91, 104-108, doi:10.1016/j.diff.2016.01.002 (2016).

39 Ciglieri, E., Ferrini, F., Boggio, E. & Salio, C. An improved method for in vitro morphofunctional analysis of mouse dorsal root ganglia. *Annals of Anatomy - Anatomischer Anzeiger* 207, 62-67, doi:10.1016/j.aanat.2016.04.032 (2016).

40 Zapiec, B., Bressel, O. C., Khan, M., Walz, A. & Mombaerts, P. Neuropilin-1 and the Positions of Glomeruli in the Mouse Olfactory Bulb. *eneuro* 3, ENEURO.0123-0116., doi:10.1523/eneuro.0123-16.2016 (2016).

41 Schueth, A., Spronck, B., Van Zandvoort, M. A. M. J. & Van Koeveringe, G. A. Computer-assisted three-dimensional tracking of sensory innervation in the murine bladder mucosa with two-photon microscopy. *Journal of Chemical Neuroanatomy* 85, 43-49, doi:10.1016/j.jchemneu.2017.06.006 (2017).

42 Sousa-Valente, J. *et al.* Inflammation of peripheral tissues and injury to peripheral nerves induce differing effects in the expression of the calcium-sensitive N-arachydonoylethanolamine-synthesizing enzyme and related molecules in rat primary sensory neurons. *Journal of Comparative Neurology* 525, 1778-1796, doi:10.1002/cne.24154 (2017).

43 Hegarty, D. M., Hermes, S. M., Yang, K. & Aicher, S. A. Select noxious stimuli induce changes on corneal nerve morphology. *Journal of Comparative Neurology* 525, 2019-2031, doi:10.1002/cne.24191 (2017).

44 Huang, T., Ohman, L. C., Clements, A. V., Whiddon, Z. D. & Krimm, R. F. Variable branching characteristics of peripheral taste neurons indicates differential convergence. *The Journal of Neuroscience* 41, JN-RM-1935-1920, doi:10.1523/jneurosci.1935-20.2021 (2021).

45 Ohman, L. C. & Krimm, R. F. Whole-Mount Staining, Visualization, and Analysis of Fungiform, Circumvallate, and Palate Taste Buds. *Journal of Visualized Experiments*, doi:10.3791/62126 (2021).

46 Van Der Woude, E. & Smid, H. M. How to escape from haller's rule: Olfactory system complexity in small and largeTrichogramma evanescensparasitic wasps. *Journal of Comparative Neurology* 524, 1876-1891, doi:10.1002/cne.23927 (2016).

47 Carle, T., Watanabe, H., Yamawaki, Y. & Yokohari, F. Organization of the antennal lobes in the praying mantis (Tenodera aridifolia). *Journal of Comparative Neurology* 525, 1685-1706, doi:10.1002/cne.24159 (2017).

48 Banerjee, S. & Chaturvedi, C. M. Testicular atrophy and reproductive quiescence in photorefractory and scotosensitive quail: Involvement of hypothalamic deep brain photoreceptors and GnRH-GnIH system. *Journal of Photochemistry and Photobiology B: Biology* 175, 254-268, doi:10.1016/j.jphotobiol.2017.09.005 (2017).

49 Lysakowski, A. *et al.* Molecular Microdomains in a Sensory Terminal, the Vestibular Calyx Ending. *Journal of Neuroscience* 31, 10101-10114, doi:10.1523/jneurosci.0521-11.2011 (2011).

50 Goldsmith, A. D., Sarin, S., Lockery, S. & Hobert, O. Developmental control of lateralized neuron size in the nematode Caenorhabditis elegans. *Neural Development* 5, 33, doi:10.1186/1749-8104-5-33 (2010).

51 Erko, M. *et al.* Micro- and nano-structural details of a spider's filter for substrate vibrations: relevance for low-frequency signal transmission. *Journal of The Royal Society Interface* 12, 20141111, doi:10.1098/rsif.2014.1111 (2015).

52 Dyer, C., Linker, C., Graham, A. & Knight, R. Specification of sensory neurons occurs through diverse developmental programs functioning in the brain and spinal cord. *Developmental Dynamics* 243, 1429-1439, doi:10.1002/dvdy.24184 (2014).

53 Jia, L. *et al.* A colour preference technique to evaluate acrylamide-induced toxicity in zebrafish. *Comparative Biochemistry and Physiology Part C: Toxicology & Pharmacology* 199, 11-19, doi:10.1016/j.cbpc.2017.01.004 (2017).

54 Wehnekamp, F., Plucińska, G., Thong, R., Misgeld, T. & Lamb, D. C. Nanoresolution real-time 3D orbital tracking for studying mitochondrial trafficking in vertebrate axons in vivo. *eLife* 8, doi:10.7554/elife.46059 (2019).

55 Takahashi, N. *et al.* Complete identification of four giant interneurons supplying mushroom body calyces in the cockroachPeriplaneta americana. *Journal of Comparative Neurology* 525, 204-230, doi:10.1002/cne.24108 (2017).

56 Schnell, B. *et al.* Processing of horizontal optic flow in three visual interneurons of the Drosophila brain. *J Neurophysiol* 103, 1646-1657, doi:10.1152/jn.00950.2009 (2010).

57 Varela, N., Avilla, J., Gemeno, C. & Anton, S. Ordinary glomeruli in the antennal lobe of male and female tortricid moth Grapholita molesta (Busck) (Lepidoptera: Tortricidae) process sex pheromone and host-plant volatiles. *Journal of Experimental Biology* 214, 637-645, doi:10.1242/jeb.047316 (2011).

58 Abrahamsson, T., Cathala, L., Matsui, K., Shigemoto, R. & Digregorio, A., David. Thin Dendrites of Cerebellar Interneurons Confer Sublinear Synaptic Integration and a Gradient of Short-Term Plasticity. *Neuron* 73, 1159-1172, doi:10.1016/j.neuron.2012.01.027 (2012).

59 Alcami, P. & Marty, A. Estimating functional connectivity in an electrically coupled interneuron network. *Proceedings of the National Academy of Sciences* 110, E4798-E4807, doi:10.1073/pnas.1310983110 (2013).

60 Trouche, S., Sasaki, M., Jennifer, Tu, T. & Reijmers, G., Leon. Fear Extinction Causes Target-Specific Remodeling of Perisomatic Inhibitory Synapses. *Neuron* 80, 1054-1065, doi:10.1016/j.neuron.2013.07.047 (2013).

61 Debertin, G. *et al.* Tyrosine hydroxylase positive perisomatic rings are formed around various amacrine cell types in the mammalian retina. *Journal of Neurochemistry* 134, 416-428, doi:10.1111/jnc.13144 (2015).

62 Riedemann, T., Schmitz, C. & Sutor, B. Immunocytochemical heterogeneity of somatostatin-expressing GABAergic interneurons in layers II and III of the mouse cingulate cortex: A combined immunofluorescence/design-based stereologic study. *Journal of Comparative Neurology* 524, 2281-2299, doi:10.1002/cne.23948 (2016).

63 Geiller, T. *et al.* Large-Scale 3D Two-Photon Imaging of Molecularly Identified CA1 Interneuron Dynamics in Behaving Mice. *Neuron* 108, 968-983.e969, doi:10.1016/j.neuron.2020.09.013 (2020).

64 Alessio, E. J. & Zhang, D.-Q. Immunostaining of Whole-Mount Retinas with the CLARITY Tissue Clearing Method. *Journal of Visualized Experiments*, doi:10.3791/62178 (2021).

65 Zikopoulos, B., John, Y. J., García-Cabezas, M. Á., Bunce, J. G. & Barbas, H. The intercalated nuclear complex of the primate amygdala. *Neuroscience* 330, 267-290, doi:10.1016/j.neuroscience.2016.05.052 (2016).

66 López, I. P. *et al.* The added value of rabies virus as a retrograde tracer when combined with dual anterograde tract-tracing. *Journal of Neuroscience Methods* 194, 21-27, doi:10.1016/j.jneumeth.2010.01.015 (2010).

67 Chai, S., Cambronne, X. A., Eichhorn, S. W. & Goodman, R. H. MicroRNA-134 activity in somatostatin interneurons regulates H-Ras localization by repressing the palmitoylation enzyme, DHHC9. *Proceedings of the National Academy of Sciences* 110, 17898-17903, doi:10.1073/pnas.1317528110 (2013).

68 Bendali, A. *et al.* Synthetic 3D diamond-based electrodes for flexible retinal neuroprostheses: Model, production and in vivo biocompatibility. *Biomaterials* 67, 73-83, doi:10.1016/j.biomaterials.2015.07.018 (2015).

69 Kastli, R. *et al.* Developmental divergence of sensory stimulus representation in cortical interneurons. *Nature Communications* 11, doi:10.1038/s41467-020-19427-z (2020).

70 Parrish, R. R., Grady, J., Codadu, N. K., Trevelyan, A. J. & Racca, C. Simultaneous profiling of activity patterns in multiple neuronal subclasses. *Journal of Neuroscience Methods* 303, 16-29, doi:10.1016/j.jneumeth.2018.03.012 (2018).

71 Biserova, N. M., Kutyrev, I. A. & Jensen, K. GABA in the nervous system of the cestodes Diphyllobothrium dendriticum (Diphyllobothriidea) and Caryophyllaeus laticeps (Caryophyllidea), with comparative analysis of muscle innervation. *J Parasitol* 100, 411-421, doi:10.1645/13-366.1 (2014).

72 Ho, T., Vessey, K. A. & Fletcher, E. L. Immunolocalization of the P2X4 receptor on neurons and glia in the mammalian retina. *Neuroscience* 277, 55-71, doi:10.1016/j.neuroscience.2014.06.055 (2014).

73 Keeley, P. W., Madsen, N. R., St John, A. J. & Reese, B. E. Programmed cell death of retinal cone bipolar cells is independent of afferent or target control. *Dev Biol* 394, 191-196, doi:10.1016/j.ydbio.2014.08.018 (2014).

74 Jiang, Y., Boll, W. & Noll, M. Pox neuro control of cell lineages that give rise to larval poly-innervated external sensory organs in Drosophila. *Developmental Biology* 397, 162-174, doi:10.1016/j.ydbio.2014.10.013 (2015).

75 Hannibal, J., Christiansen, A. T., Heegaard, S., Fahrenkrug, J. & Kiilgaard, J. F. Melanopsin expressing human retinal ganglion cells: Subtypes, distribution, and intraretinal connectivity. *Journal of Comparative Neurology* 525, 1934-1961, doi:10.1002/cne.24181 (2017).

76 Takács, S. *et al.* Post mortem single-cell labeling with DiI and immunoelectron microscopy unveil the fine structure of kisspeptin neurons in humans. *Brain Structure and Function* 223, 2143-2156, doi:10.1007/s00429-018-1610-8 (2018).

77 Hartveit, E. *et al.* AMPA receptors at ribbon synapses in the mammalian retina: kinetic models and molecular identity. *Brain Structure and Function* 223, 769-804, doi:10.1007/s00429-017-1520-1 (2018).

78 Santos, R. A. *et al.* DSCAM differentially modulates pre- and postsynaptic structural and functional central connectivity during visual system wiring. *Neural Development* 13, doi:10.1186/s13064-018-0118-5 (2018).

79 Choi, J.-H., Law, M.-Y., Chien, C.-B., Link, B. A. & Wong, R. O. In vivo development of dendritic orientation in wild-type and mislocalized retinal ganglion cells. *Neural Development* 5, 29, doi:10.1186/1749-8104-5-29 (2010).

80 Rahman, T. *et al.* Cofilin rods and aggregates concur with tau pathology and the development of Alzheimer's disease. *J Alzheimers Dis* 42, 1443-1460, doi:10.3233/JAD-140393 (2014).

81 Salamanca, L. *et al.* MIC‐MAC: An automated pipeline for high‐throughput characterization and classification of three‐dimensional microglia morphologies in mouse and human postmortem brain samples. *Glia* 67, 1496-1509, doi:10.1002/glia.23623 (2019).

82 Jawaid, S. *et al.* Alterations in CA1 hippocampal synapses in a mouse model of fragile X syndrome. *Glia* 66, 789-800, doi:10.1002/glia.23284 (2018).

83 Dibaj, P. *et al.* Long-lasting post-mortem activity of spinal microglia in situ in mice. *Journal of Neuroscience Research*, NA-NA, doi:10.1002/jnr.22402 (2010).

84 Masuda, T., Croom, D., Hida, H. & Kirov, S. A. Capillary blood flow around microglial somata determines dynamics of microglial processes in ischemic conditions. *Glia* 59, 1744-1753, doi:10.1002/glia.21220 (2011).

85 Ertürk, A. *et al.* Three-dimensional imaging of the unsectioned adult spinal cord to assess axon regeneration and glial responses after injury. *Nature Medicine* 18, 166-171, doi:10.1038/nm.2600 (2012).

86 Jiang, S. X., Slinn, J., Aylsworth, A. & Hou, S. T. Vimentin participates in microglia activation and neurotoxicity in cerebral ischemia. *Journal of Neurochemistry* 122, 764-774, doi:10.1111/j.1471-4159.2012.07823.x (2012).

87 Sosa, R. A., Murphey, C., Ji, N., Cardona, A. E. & Forsthuber, T. G. The Kinetics of Myelin Antigen Uptake by Myeloid Cells in the Central Nervous System during Experimental Autoimmune Encephalomyelitis. *The Journal of Immunology* 191, 5848-5857, doi:10.4049/jimmunol.1300771 (2013).

88 Plog, B. A. *et al.* A novel technique for morphometric quantification of subarachnoid hemorrhage-induced microglia activation. *Journal of Neuroscience Methods* 229, 44-52, doi:10.1016/j.jneumeth.2014.04.001 (2014).

89 Bayerl, S. H. *et al.* Time lapsein vivomicroscopy reveals distinct dynamics of microglia-tumor environment interactions-a new role for the tumor perivascular space as highway for trafficking microglia. *Glia* 64, 1210-1226, doi:10.1002/glia.22994 (2016).

90 Xu, N. *et al.* Fast free-of-acrylamide clearing tissue (FACT)—an optimized new protocol for rapid, high-resolution imaging of three-dimensional brain tissue. *Scientific Reports* 7, doi:10.1038/s41598-017-10204-5 (2017).

91 Dando, S. J., Kazanis, R., Chinnery, H. R. & Mcmenamin, P. G. Regional and functional heterogeneity of antigen presenting cells in the mouse brain and meninges. *Glia* 67, 935-949, doi:10.1002/glia.23581 (2019).

92 Venkateswarlu, K. *et al.* Three‐dimensional imaging and quantification of real‐time cytosolic calcium oscillations in microglial cells cultured on electrospun matrices using laser scanning confocal microscopy. *Biotechnology and Bioengineering* 117, 3108-3123, doi:10.1002/bit.27465 (2020).

93 Singaravelu, J., Zhao, L., Fariss, R. N., Nork, T. M. & Wong, W. T. Microglia in the primate macula: specializations in microglial distribution and morphology with retinal position and with aging. *Brain Structure and Function* 222, 2759-2771, doi:10.1007/s00429-017-1370-x (2017).

94 Fujioka, H., Kakehashi, C., Funabashi, T. & Akema, T. Immunohistochemical evidence for the relationship between microglia and GnRH neurons in the preoptic area of ovariectomized rats with and without steroid replacement. *Endocr J* 60, 191-196, doi:10.1507/endocrj.ej12-0280 (2013).

95 Grinberg, Y. Y., Dibbern, M. E., Levasseur, V. A. & Kraig, R. P. Insulin-like growth factor-1 abrogates microglial oxidative stress and TNF-alpha responses to spreading depression. *J Neurochem* 126, 662-672, doi:10.1111/jnc.12267 (2013).

96 Zhang, Y. *et al.* Image processing methods to elucidate spatial characteristics of retinal microglia after optic nerve transection. *Scientific Reports* 6, 21816, doi:10.1038/srep21816 (2016).

97 Althammer, F. *et al.* Three-dimensional morphometric analysis reveals time-dependent structural changes in microglia and astrocytes in the central amygdala and hypothalamic paraventricular nucleus of heart failure rats. *Journal of Neuroinflammation* 17, doi:10.1186/s12974-020-01892-4 (2020).

98 Xiao, H., Li, Y., Du, J. & Mosig, A. Ct3d: tracking microglia motility in 3D using a novel cosegmentation approach. *Bioinformatics* 27, 564-571, doi:10.1093/bioinformatics/btq691 (2011).

99 Pihlaja, R. *et al.* Transplanted astrocytes internalize deposited β-amyloid peptides in a transgenic mouse model of Alzheimer's disease. *Glia* 56, 154-163, doi:10.1002/glia.20599 (2008).

100 Tavares, G. *et al.* Employing an open-source tool to assess astrocyte tridimensional structure. *Brain Structure and Function* 222, 1989-1999, doi:10.1007/s00429-016-1316-8 (2017).

101 Chvátal, A., Anděrová, M. & Kirchhoff, F. Three-dimensional confocal morphometry ? a new approach for studying dynamic changes in cell morphology in brain slices. *Journal of Anatomy* 210, 671-683, doi:10.1111/j.1469-7580.2007.00724.x (2007).

102 Miller, S. J. & Rothstein, J. D. Astroglia in Thick Tissue with Super Resolution and Cellular Reconstruction. *PLoS One* 11, e0160391, doi:10.1371/journal.pone.0160391 (2016).

103 Wang, R., Seifert, P. & Jakobs, T. C. Astrocytes in the Optic Nerve Head of Glaucomatous Mice Display a Characteristic Reactive Phenotype. *Invest Ophthalmol Vis Sci* 58, 924-932, doi:10.1167/iovs.16-20571 (2017).

104 Li, D. *et al.* Lack of Evidence for Vesicular Glutamate Transporter Expression in Mouse Astrocytes. *Journal of Neuroscience* 33, 4434-4455, doi:10.1523/jneurosci.3667-12.2013 (2013).

105 Paul, D. *et al.* Cell-selective knockout and 3D confocal image analysis reveals separate roles for astrocyte-and endothelial-derived CCL2 in neuroinflammation. *Journal of Neuroinflammation* 11, 10, doi:10.1186/1742-2094-11-10 (2014).

106 Ugbode, C. I., Hirst, W. D. & Rattray, M. Neuronal influences are necessary to produce mitochondrial co‐localization with glutamate transporters in astrocytes. *Journal of Neurochemistry* 130, 668-677, doi:10.1111/jnc.12759 (2014).

107 Förtsch, C. *et al.* Changes in Astrocyte Shape Induced by Sublytic Concentrations of the Cholesterol-Dependent Cytolysin Pneumolysin Still Require Pore-Forming Capacity. *Toxins* 3, 43-62, doi:10.3390/toxins3010043 (2011).

108 Quesseveur, G., Fouquier d'Herouel, A., Murai, K. K. & Bouvier, D. S. A Specialized Method to Resolve Fine 3D Features of Astrocytes in Nonhuman Primate (Marmoset, Callithrix jacchus) and Human Fixed Brain Samples. *Methods Mol Biol* 1938, 85-95, doi:10.1007/978-1-4939-9068-9_6 (2019).

109 Testen, A., Kim, R. & Reissner, K. J. High‐Resolution Three‐Dimensional Imaging of Individual Astrocytes Using Confocal Microscopy. *Current Protocols in Neuroscience* 91, doi:10.1002/cpns.92 (2020).

110 Bagheri, M. *et al.* Amyloid Beta1-40-Induced Astrogliosis and the Effect of Genistein Treatment in Rat: A Three-Dimensional Confocal Morphometric and Proteomic Study. *PLoS ONE* 8, e76526, doi:10.1371/journal.pone.0076526 (2013).

111 Walsh, N., Valter, K. & Stone, J. Cellular and Subcellular Patterns of Expression of bFGF and CNTF in the Normal and Light Stressed Adult Rat Retina. *Experimental Eye Research* 72, 495-501, doi:10.1006/exer.2000.0984 (2001).

112 Barton, E. A. *et al.* Binge alcohol alters exercise-driven neuroplasticity. *Neuroscience* 343, 165-173, doi:10.1016/j.neuroscience.2016.11.041 (2017).

113 Williams, P. R. *et al.* In Vivo Development of Outer Retinal Synapses in the Absence of Glial Contact. *Journal of Neuroscience* 30, 11951-11961, doi:10.1523/jneurosci.3391-10.2010 (2010).

114 Kulkarni, P. M. *et al.* Quantitative 3-D analysis of GFAP labeled astrocytes from fluorescence confocal images. *J Neurosci Methods* 246, 38-51, doi:10.1016/j.jneumeth.2015.02.014 (2015).

115 Bagheri, M., Rezakhani, A., Roghani, M., Joghataei, M. T. & Mohseni, S. Protocol for Three-dimensional Confocal Morphometric Analysis of Astrocytes. *Journal of Visualized Experiments*, doi:10.3791/53113 (2015).

116 Tomita, M., Tomita, Y., Unekawa, M., Toriumi, H. & Suzuki, N. Oscillating neuro-capillary coupling during cortical spreading depression as observed by tracking of FITC-labeled RBCs in single capillaries. *Neuroimage* 56, 1001-1010, doi:10.1016/j.neuroimage.2011.02.078 (2011).

117 Roqué, P. J., Guizzetti, M., Giordano, G. & Costa, L. G. in *Methods in Molecular Biology* 361-390 (Humana Press, 2011).

118 Lind, B. L., Brazhe, A. R., Jessen, S. B., Tan, F. C. C. & Lauritzen, M. J. Rapid stimulus-evoked astrocyte Ca2+ elevations and hemodynamic responses in mouse somatosensory cortex in vivo. *Proceedings of the National Academy of Sciences* 110, E4678-E4687, doi:10.1073/pnas.1310065110 (2013).

119 Smith, G. S. T. *et al.* Classical 18.5-and 21.5-kDa isoforms of myelin basic protein inhibit calcium influx into oligodendroglial cells, in contrast to golli isoforms. *Journal of Neuroscience Research* 89, 467-480, doi:10.1002/jnr.22570 (2011).

120 Azaripour, A. *et al.* Three-dimensional histochemistry and imaging of human gingiva. *Scientific Reports* 8, doi:10.1038/s41598-018-19685-4 (2018).

121 Mourik, M. J. *et al.* in *Methods in Cell Biology* 71-92 (Elsevier, 2014).

122 Song, W. *et al.* Three-dimensional morphometric comparison of normal and apoptotic endothelial cells based on laser scanning confocal microscopy observation. *Microscopy Research and Technique* 76, 1154-1162, doi:10.1002/jemt.22279 (2013).

123 Khuon, S. *et al.* Myosin light chain kinase mediates transcellular intravasation of breast cancer cells through the underlying endothelial cells: a three-dimensional FRET study. *Journal of Cell Science* 123, 431-440, doi:10.1242/jcs.053793 (2010).

124 Ueki, Y., Uda, Y., Sakamoto, N. & Sato, M. Measurements of strain on single stress fibers in living endothelial cells induced by fluid shear stress. *Biochemical and Biophysical Research Communications* 395, 441-446, doi:10.1016/j.bbrc.2010.04.051 (2010).

125 Esch, M. B., Post, D. J., Shuler, M. L. & Stokol, T. Characterization of In Vitro Endothelial Linings Grown Within Microfluidic Channels. *Tissue Engineering Part A* 17, 2965-2971, doi:10.1089/ten.tea.2010.0371 (2011).

126 Schulz, M. M. P. *et al.* Phenotype-based high-content chemical library screening identifies statins as inhibitors of in vivo lymphangiogenesis. *Proceedings of the National Academy of Sciences* 109, E2665-E2674, doi:10.1073/pnas.1206036109 (2012).

127 Nikolaisen, J. *et al.* Automated Quantification and Integrative Analysis of 2D and 3D Mitochondrial Shape and Network Properties. *PLoS ONE* 9, e101365, doi:10.1371/journal.pone.0101365 (2014).

128 Stiles, J. M. *et al.* Morphological restriction of human coronary artery endothelial cells substantially impacts global gene expression patterns. *The FEBS Journal* 280, 4474-4494, doi:10.1111/febs.12410 (2013).

129 Martin, M. *et al.* PP2A regulatory subunit Balpha controls endothelial contractility and vessel lumen integrity via regulation of HDAC7. *EMBO J* 32, 2491-2503, doi:10.1038/emboj.2013.187 (2013).

130 Blacher, S. *et al.* Cell Invasion in the Spheroid Sprouting Assay: A Spatial Organisation Analysis Adaptable to Cell Behaviour. *PLoS ONE* 9, e97019, doi:10.1371/journal.pone.0097019 (2014).

131 Ong, L.-L. S., Dauwels, J., Ang, M. H. & Asada, H. H. A Bayesian filtering approach to incorporate 2D/3D time-lapse confocal images for tracking angiogenic sprouting cells interacting with the gel matrix. *Medical Image Analysis* 18, 211-227, doi:10.1016/j.media.2013.10.008 (2014).

132 Annamdevula, N. S. *et al.* Spectral imaging of FRET‐based sensors reveals sustained cAMP gradients in three spatial dimensions. *Cytometry Part A* 93, 1029-1038, doi:10.1002/cyto.a.23572 (2018).

133 Zohar, B. *et al.* Multi-flow channel bioreactor enables real-time monitoring of cellular dynamics in 3D engineered tissue. *Communications Biology* 2, doi:10.1038/s42003-019-0400-z (2019).

134 Kaushik, G. *et al.* Quantitative Label‐Free Imaging of 3D Vascular Networks Self‐Assembled in Synthetic Hydrogels. *Advanced Healthcare Materials* 8, 1801186, doi:10.1002/adhm.201801186 (2019).

135 Cafaro, T. A. *et al.* On the Cornea of Healthy Merino Sheep: A Detailed Ex Vivo Confocal, Histological and Ultrastructural Study. *Anatomia, Histologia, Embryologia* 44, 247-254, doi:10.1111/ahe.12131 (2015).

136 Tan, P. E. Z., Yu, P. K., Cringle, S. J., Morgan, W. H. & Yu, D.-Y. Regional heterogeneity of endothelial cells in the porcine vortex vein system. *Microvascular Research* 89, 70-79, doi:10.1016/j.mvr.2013.06.004 (2013).

137 Boselli, F., Goetz, J. G., Charvin, G. & Vermot, J. in *Methods in Cilia & Flagella* 161-173 (Elsevier, 2015).

138 An, D., Yu, P., Freund, K. B., Yu, D.-Y. & Balaratnasingam, C. Three-Dimensional Characterization of the Normal Human Parafoveal Microvasculature Using Structural Criteria and High-Resolution Confocal Microscopy. *Investigative Opthalmology & Visual Science* 61, 3, doi:10.1167/iovs.61.10.3 (2020).

139 Sokic, S. *et al.* Label-free nondestructive imaging of vascular network structure in 3D culture. *Microvascular Research* 92, 72-78, doi:10.1016/j.mvr.2014.01.003 (2014).

140 Cain, R. J., D’Água, B. B. & Ridley, A. J. in *Methods in Molecular Biology* 167-190 (Humana Press, 2011).

141 Zeitlin, B. D., Dong, Z. & Nör, J. E. RAIN-Droplet: a novel 3D in vitro angiogenesis model. *Laboratory Investigation* 92, 988-998, doi:10.1038/labinvest.2012.77 (2012).

142 Zhong, W. *et al.* Prox1-GFP/Flt1-DsRed transgenic mice: an animal model for simultaneous live imaging of angiogenesis and lymphangiogenesis. *Angiogenesis* 20, 581-598, doi:10.1007/s10456-017-9572-7 (2017).

143 Ghosh, M. *et al.* Specific Visualization of Nitric Oxide in the Vasculature with Two-Photon Microscopy Using a Copper Based Fluorescent Probe. *PLoS ONE* 8, e75331, doi:10.1371/journal.pone.0075331 (2013).

144 Errede, M., Girolamo, F. & Virgintino, D. in *Methods in Molecular Biology* 143-150 (Springer US, 2021).

145 Nikolajsen, G. N., Jensen, M. S. & West, M. J. A zinc fixative for 3D visualization of cerebral capillaries and pericytes. *Journal of Neuroscience Methods* 257, 1-6, doi:10.1016/j.jneumeth.2015.09.016 (2016).

146 Faye, N. *et al.* Antitumoral Effect of Mural Cells Assessed With High-Resolution MRI and Fluorescence Microscopy. *American Journal of Roentgenology* 205, W11-W18, doi:10.2214/ajr.14.13680 (2015).

147 Held, K. F. & Dostmann, W. R. in *Methods in Molecular Biology* 131-145 (Humana Press, 2013).

148 Kan, C., Yip, K.-P. & Yang, H. Two-Phase Greedy Pursuit Algorithm for Automatic Detection and Characterization of Transient Calcium Signaling. *IEEE Journal of Biomedical and Health Informatics* 19, 687-697, doi:10.1109/jbhi.2014.2312293 (2015).

149 Bankhead, P., Scholfield, C. N., Curtis, T. M. & McGeown, J. G. Detecting Ca2+ sparks on stationary and varying baselines. *Am J Physiol Cell Physiol* 301, C717-728, doi:10.1152/ajpcell.00032.2011 (2011).

150 Halaidych, O. V., Mummery, C. L. & Orlova, V. V. Quantifying Ca2+ signaling and contraction in vascular pericytes and smooth muscle cells. *Biochemical and Biophysical Research Communications* 513, 112-118, doi:10.1016/j.bbrc.2019.03.143 (2019).
